# Game-based situation awareness training for child and adult cyclists

**DOI:** 10.1098/rsos.160823

**Published:** 2017-03-22

**Authors:** Esko Lehtonen, Jasmiina Airaksinen, Kaisa Kanerva, Anna Rissanen, Riikka Ränninranta, Veera Åberg

**Affiliations:** 1 Traffic Research Unit, Cognitive Science, Faculty of Arts, University of Helsinki, Helsinki, Finland; 2 Department of Psychology and Logopedics, Faculty of Medicine, University of Helsinki, Helsinki, Finland

**Keywords:** situational awareness, hazard perception, bicycling, serious games, eye movements, working memory

## Abstract

Safe cycling requires situation awareness (SA), which is the basis for recognizing and anticipating hazards. Children have poorer SA than adults, which may put them at risk. This study investigates whether cyclists' SA can be trained with a video-based learning game. The effect of executive working memory on SA was also studied. Thirty-six children (9–10 years) and 22 adults (21–48 years) played the game. The game had 30 video clips filmed from a cyclist's perspective. Each clip was suddenly masked and two or three locations were presented. The player's task was to choose locations with a potential hazard and feedback was given for their answers. Working memory capacity (WMC) was tested with a counting span task. Children's and adults' performance improved while playing the game, which suggests that playing the game trains SA. Adults performed better than children, and they also glanced at hazards more while the video was playing. Children expectedly had a lower WMC than adults, but WMC did not predict performance within the groups. This indicates that SA does not depend on WMC when passively viewing videos.

## 1. Introduction

Cycling is a common and growing mode of transportation especially in urban areas. While the overall health benefits of cycling outweigh the negative effects of crashes, injuries and fatalities are a considerable problem [1–3]. Children who cycle are a particular risk group in traffic. In the European Union between 2008 and 2010, 59% of children who were non-fatally injured in traffic were cyclists [4]. In Finland between 1998 and 2007, 47% of injuries among 0–14 year olds requiring hospital care occurred in cycling [5]. Children are often involved in crashes when they have entered the roadway from driveways, sidewalks or mid-block in a manner which suggests that they have failed to perform a visual search and anticipate oncoming cars [6]. In other words, children's poor situation awareness (SA) may contribute to their risk for crashes [7,8].

### 1.1. Situation awareness and hazard perception

SA is an actor's (e.g. a cyclist's) representation of the situation relative to their goals, and that representation is used to guide decisions and actions. According to Endsley's [9] formulation, SA consists of three levels: (i) perceiving the relevant elements in the environment, (ii) comprehending the elements with respect to the situation and one's own goals, and (iii) predicting how the situation develops.

Hazard perception (HP) is defined as the ability to perceive and anticipate situations in traffic which have a high probability of leading to a crash [10] and can be understood as SA under hazardous traffic situations [11,12]. The most important distinction between HP and SA is that the HP tasks measure reactions to hazards, [11,13], whereas SA tasks probe for the presence of elements, ask for interpretation of the situation, or ask ‘what happens next’ [14–17]. This means that HP tasks can be affected by cognitive processes not used in pure SA tasks, like an individual's interpretation of a hazard, including their perceived level of risk and their criteria for marking something as a hazard.

Although any road user or environmental element which may cause a crash is a hazard, not all hazards are equal when considering HP. A simple but useful typology is to classify hazards as *overt* and *covert* hazards, which can also be either *latent* or *acute* [18]. Overt hazards are fully visible, like a pedestrian who is approaching a zebra crossing. Covert hazards are hidden, e.g. a parked van which may hide a child stepping into the road. When a hazard is acute, it requires an immediate reaction to avoid a collision, while for a latent hazard, there are cues which can be used to predict the potential hazard. It has been suggested that the main source of individual differences in HP skill is the ability to use cues to predict latent hazards [12,18]. This means that individuals with good HP skills must be able to acquire high level SA of the situation.

### 1.2. Effects of age and experience on hazard perception

The acquisition of better mental models of traffic situations through experience has been identified as one of the key factors contributing to individual differences in HP [11,19]. HP as a function of experience has often been investigated in car driving in adults or adolescents with a driving licence or who are still learner drivers. (For HP and motorcycling see e.g. [20,21].) Compared with experienced drivers, novice drivers have more difficulties in recognizing latent hazards and react slower [11,13,16–18]. Cycling experience may have a similar effect in a bicycling context [22].

Experienced drivers visually search for hazard-related cues more than novices. They fixate on a larger proportion of the cues, have larger dwell times on cues and make their first fixation on cues earlier than novices [10,23,24]. It is common for novice drivers to focus on new, unusual, dangerous or complicated stimuli and to be unable to divide their attention as well as experienced drivers [25,26]. More extensive visual scanning of hazards by experienced drivers typically results in a larger horizontal spread of eye movements [27,28].

Children's performance in video-based or simulated HP or SA tasks is poorer than adults' [7,8,29,30]. Covert hazards, like an approaching vehicle which is occluded by road curviness or parked cars, are especially challenging for children to notice [7,8]. This can be partly due to their lack of experience in traffic, but their ongoing perceptual, cognitive and motor development play a major role [31]. Compared with adults, children have less developed attentional skills [32–34], which can affect their ability to acquire SA. They are also less sensitive to the visual looming effect which makes it harder for them to spot an approaching car [35]. In dual-task settings, they often prioritize motor tasks over perceptual or cognitive tasks [36,37] and poorly coordinate their movements relative to the other dynamically moving road users [38,39].

### 1.3. Training

HP, as measured by a video-based test, has been linked to crash involvement in a prospective setting [40]. Consequently, it is very relevant to develop ways to train HP or SA, and HP training programmes designed for young novice drivers have shown promising results [41]. Interactive computer-based training in particular has improved young drivers' visual scanning of relevant locations both in simulator studies and on the road [42,43]. Recently, there has been an interest in training children's HP/SA using computer-based or simulated environments without exposing children to actual hazards [44,45].

Training programmes which are designed to be played as games can make such training more motivating for children. Lehtonen *et al*. [8] tested a video-based game where the players had to point out relevant targets while the video was playing. Points were awarded for hits and feedback was given for misses. The game training shortened answer latencies in SA tests for both adults and children who played but did not improve their sensitivity to targets.

### 1.4. Research questions and hypotheses

Earlier research demonstrates that experienced adults are more skilled in anticipating hazards than novices and that children are less skilled than adults. In addition, previous research indicates that HP can be trained. However, only a few studies have focused on training among children. Recently, the contribution of working memory capacity (WMC) [46] on HP has been investigated among adults [47,48], but no studies linking SA and WMC among children have been made.

In this study, a group of children and a group of adults played a learning game designed to assess and train their SA in the context of cycling. In the game, the participants watched videos filmed from a cyclist's perspective, and they were told to keep an eye on the other road users and occlusions where someone could emerge. Each video was suddenly masked and the participants were asked to locate these targets by selecting among multiple locations. Targets were selected so that they were overt or covert latent hazards. The game was a gamified modification of the SA test used in Lehtonen *et al*. [8], modified by giving feedback and points for the answers.

The primary aim of this study was to investigate if the players' SA improves while they play the game. In addition, we expected that adults would have a better SA than children, and more experienced adult cyclists would have a better SA than the less experienced adults. To complement the behavioural data from the game, eye movements were analysed. Anticipatory glances to 10 overt hazards were analysed for both groups. Better performance in the game was expected to be positively correlated with looking more often and earlier at the hazards.

The secondary aim of the study was to investigate if a larger WMC is associated with better SA. Previous research among adults has indicated that low WMC affects HP tasks negatively, but only in dual-tasking conditions (e.g. while simultaneously steering) [47,48]. However, adults have both higher WMC and more experience (better domain knowledge) which may help them to overcome the limitations in WMC [49,50]. Thus, it is possible that even the acquisition and maintenance of SA without any dual-task components could reach the limits of children's WMC.

## 2. Material and methods

### 2.1. Participants

Child participants were recruited from a local primary school. In total, 45 children were invited and 36 participated. Adults were recruited through the university student e-mail lists. There were 36 children (15 girls, 21 boys), aged 9 to 10 years (*M* = 9.1, Mdn = 9) and 22 adults (17 females, 5 males), aged 21 to 48 years (*M* = 26.3, Mdn = 24). Adults were categorized as experienced cyclists (*N* = 14) or inexperienced cyclists (*N* = 8) based on their self-reported cycling habits. Experienced cyclists were required to cycle at least a few times per week in their cycling season (12 cycled daily or almost daily, two cycled a few times a week). Inexperienced cyclists cycled at most a few times per month in their cycling season (three never cycled, two cycled rarely, three cycled a few times per month).

### 2.2. Video clips

The video clips of the game were filmed in Helsinki, Finland, and depicted naturalistic cycling by an active cyclist. Videos were filmed by using a GoPro Hero HD1 camera that was attached to the head tube of a bicycle at a height of 90 cm from the ground. This mounting position provides maximum stability and was approximately at the level of a cycling child's eyes. Elastic padding was used in the joint to remove high frequency vibration. The field of view of the camera was 170°.

First, the cyclist extracted clips from the video footage based on his own judgement. Then, 30 video clips were selected for the game by the researchers, and an additional three clips were used for practice. The duration of the clips in the game was between 4.81 s and 24.19 s (*M* = 11.47 s, Mdn = 10.71 s, s.d. = 5.08 s). The clips were chosen so that they included a situation where it was possible to choose two or three locations of which one or two had a target present and the rest were empty. (See electronic supplementary material, table S1 for details.) The targets were either overt, like other road users, or covert, such as bus stops and corners of buildings. Overt targets were chosen so that the road users were either on an intersecting path with the cyclists or could change the course and end up on an intersecting path. Covert targets were such environmental objects behind which someone could suddenly emerge on an intersecting path. Thus overt and covert targets could be categorized as latent hazards [18]. Locations without a target were marked as areas that did not contain any notable objects or other possible hazards, for example plain asphalt or the wall of a building. In total, there were 33 locations with an overt target, 12 with a covert target and 34 without a target.

### 2.3. Game

**Figure 1.**
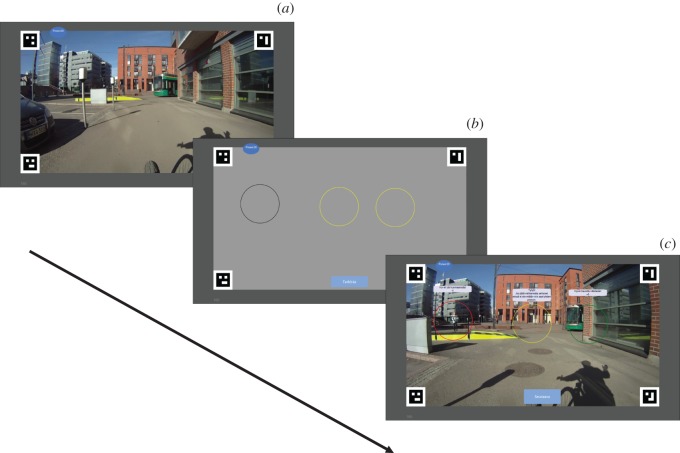
The progress of the game. (*a*) The player watches a video from a cyclist's perspective. At a predefined time the video is stopped and masked. (*b*) Two to three locations are highlighted with a black circle in the masked video and the player's task is to select those locations where there was either an overt or covert target present at the moment when the video was stopped and masked. In selection, the circle turns to yellow. The player can change their answers until pressing the button ‘Check’ at the bottom of the video. (*c*) Finally, the mask is removed and feedback is given, and the player can proceed to the next video by pressing a button. Correctly selected locations are shown with green, incorrectly selected empty location with yellow, and incorrectly unselected with red. Textual feedback with points gained/lost is given for each location.

The game started with three practice videos and then continued with 30 videos in a randomized order. Participants were told to keep an eye on the other road users (overt targets) and occlusions where someone could emerge (covert targets). Each video was shown until the predefined stopping situation where the video was masked and two to three locations were presented. Each location was highlighted with a circle. The player's task was to choose only those locations which contained either an overt or covert target at the moment when the video was masked (figure 1). The task in the game is essentially an adaption of the Situation Awareness Global Assessment Technique (SAGAT) for measuring SA [14].

After players answered, the game displayed the correct answers, gave feedback and increased or reduced points. By choosing a location with a target the player gained five points, and by failing to select a location with a target the player lost five points. Correctly leaving a location without a target unselected also gave one point, but selecting an empty location did not give or take points. The motivation of the points system was to reward correct selection of locations with a target, but at the same time to give the message that it is never wrong to play it safe and suspect that there might be something. However, it was still necessary to reward players for not selecting empty locations, because otherwise selecting all locations would have been an optimal strategy for gaining points.

Along with points, players also received audio and textual feedback: ‘Well detected!’, ‘Oh no, you missed a target!’, ‘Well done! That was empty.’, or ‘If you don't select empty locations you gain one point’. If a target was covert, it was mentioned: ‘That is a view blocker’. The points collected were visible at all times in the upper left corner of the screen. The points collected could never fall below zero.

### 2.4. Game performance measures

Performance in the game can be characterized by the points gained. However, because this is dependent on the specific scoring system of the game, the analyses will concentrate on responses given. Responses in the game were binary (either present or not present). Over a collection of such responses, it is possible to calculate the accuracy as the percentage of locations correctly selected or unselected out of all the locations. Other measures include hit rate, as the percentage of selected locations with a target, and the correct rejection rate, as the percentage of unselected locations without a target. The complements of these are the miss rate (100% – hit rate) and the false alarm rate (100% – correct rejection rate).

Accuracy will be used as the main measure to characterize learning. However, accuracy can improve for two reasons. The participants may either become better able to discriminate between locations with or without a target, or they may change their response bias, i.e. the propensity to select a location regardless of its content. If the participants were conservative in their answers (selecting fewer locations with a target), their accuracy may improve if they start selecting more locations at random. This is especially true in the current situation, where there were more locations with a target than without. Using signal detection theory (SDT) [51–53], it is possible to discriminate between these two effects.

In SDT, it is assumed that participants estimate the degree they think that a location contains a target. This estimate is modelled as a unidimensional decision variable. The participant then compares the estimate to their selected criterion. If the decision variable is higher than the criterion, the participant will select the location; otherwise, the location is left unselected. The decision variable values for locations without a target (‘noise’) and with a target (‘signal’) are modelled as two normal distributions which typically overlap. When there is overlap, it is not possible to answer correctly every time.

Sensitivity index *d*′ was calculated as [53, eqn 2.4]

2.1 $$d^{\prime }=\Phi^{-1}(\mathrm{hit}\;\mathrm{rate})-\Phi^{-1}(\mathrm{false}\;\mathrm{alarm}\;\mathrm{rate}),$$

where the hit rate and the false alarm rate are proportions and *Φ*^−1^ is the ‘inverse phi’ function, which converts probabilities into *z*-scores. *d*′ denotes the distance between the peaks of the signal and noise distributions in standard deviation units. Higher values of *d*′ indicate better ability to discriminate between the signal and noise. *d*′ increases when the hit rate increases or the false alarm rate decreases.

Response bias was calculated as the difference between the observed criterion and the optimal criterion.^1^ Negative values indicate a bias to select locations, and positive values indicate a bias to not select them.

The observed criterion was calculated as [53, eqn 2.3]:

2.2 $$\lambda_{\mathrm{obs}}=\Phi^{-1}(\mathrm{false}\;\mathrm{alarm}\;\mathrm{rate}).$$

The optimal criterion given the sensitivity *d*′ and the probability of a location with a target *s* was calculated as [53, eqn 2.16]:

2.3 $$\lambda_{\mathrm{optimal}}\;=0.5\times d^{\prime }-\left[\frac{\mathrm{logit}\;(s)}{d^{\prime }}\right].$$

Because the calculation of SDT measures does not allow zero false alarm rates, the log-linear rule was applied [54]. In total, 0.5 was added to the number of hits and false alarms and 1 to the number of signal and noise trials.

We also calculated the response time for each clip. The response time was the time in seconds from stopping and masking the video until the participant pressed a button to check their answers.

### 2.5. Eye movement analysis

Eye movements to 10 targets in different videos were analysed using the area of interest method. Area of interest was defined dynamically from the moment the target was first identifiable until the video was masked. These 10 targets were chosen so that their areas of interest did not overlap with other targets in the video at any moment. The targets were not located at the centre of the screen so that glances to them could be distinguished from the tendency to look in the direction of the movement.

Eye movements were measured from all participants but usable data were gathered from 32 children, 12 experienced adults and seven inexperienced adults. Participants' data were excluded if the accuracy tests performed before and after a recording session indicated that the calibration was off or that it had drifted too much during the recording. An average error of more than two degrees in a nine point accuracy test was held as an exclusion criteria. In addition, if visual inspection of the eye movement visualizations indicated that tracking did not function on some part of the screen or was strongly biased, the participant's data were excluded. The visual inspection of the data was performed before any eye movement analyses were performed. Additionally, data from 22 clips were excluded because of too many missing data points. In some clips, the eye camera of the tracker could not hold up a satisfactory frame rate. Excluded clips had an average sample rate of less than 5 Hz.

### 2.6. Working memory capacity test

WMC was measured with the counting span task [55]. The computerized version used is described in Kanerva *et al*. [56]. In the counting span task, a participant is presented with an array of dots on the computer screen, required to count the number of the dots (varied between 2 and 8), and recall the tally until prompted to report it. The participant responds by giving the recalled digits using a keyboard presented on the touch screen. The difficulty of the task is varied by the number of arrays presented before prompting the response, which forces the participant to count the dots and memorize the previous tallies at the same time.

The current task started by presenting two arrays of dots (two tallies to remember) three times. Then the amount of the arrays was increased by one and the task was again repeated three times. The number of arrays was increased until there were eight tallies to remember. However, if a participant failed to recall the correct set of tallies three times in row, the task was stopped before reaching the maximum number of arrays.

Task performance was quantified using partial-credit unit scoring (PCU), which is calculated as an average of the proportion of tallies that were recalled correctly [46]. PCU score of 0 means that the participant did not get any of the tallies right, and 1 means that they reported all tallies from all the arrays exactly right. There were at maximum (2 + 3 + 4 + 5 + 6 + 7 + 8) × 3 = 105 tallies to remember. The data from one adult participant were lost due to an error in saving.

### 2.7. Procedure

The game and the WMC test were presented with a touch screen (23^″^ Acer T232HL) connected to a computer (HP EliteBook 850 G2). The video clips were shown on a 44 × 24.5 cm large area in the game.

Eye movements were measured with a prototype version (year 2014) of the Pupil Lab's head-mounted eye tracker. The tracker had a world camera and eye movement camera for the right eye. The world camera had a frame rate of 24 fps. The nominal frame rate of the eye camera was 30 fps but the effective frame rate varied between 10 and 30 fps.^2^ Calibration of the eye tracker was carried out after the practice clips and recalibration was done at the midpoint of the game. The accuracy of the calibration was tested before and after recordings.

Before the actual game, the player practised with three video clips and got instructions from one of the researchers. After the practice, the players were able play the game at their own pace. Playing the game took 15–20 min, of which 5 min 44 s were spent watching the videos.

After the game the participants performed the counting span task. Finally, the adult players answered a short questionnaire on their background information (e.g. how often and how much they cycle). For children, a researcher completed a modified questionnaire based on the answers of the child. Questionnaires also contained questions asking players to give feedback on the game.

### 2.8. Statistical analyses

Welch's unequal variances *t*-test was used when comparing two samples because equal variance could not be typically assumed. Learning was analysed with repeated measures ANOVAs after dividing the game clips into three phases. Linear regression models were used to analyse the relationship of accuracy between WMC and eye movement parameters. Because the accuracy was a proportion, logit transformation, ln(accuracy/(1 − accuracy)), was performed before the regressions. A significance level of *p* < 0.05 was used. Effect sizes are reported using Cohen's *d*, partial *η*^2^, and adjusted coefficient of determination, depending on the analysis.

## 3. Results

The children and adults were compared by calculating their overall accuracy in the game. Children had a lower accuracy (*M* = 72%, s.d. = 7) than adults (*M* = 83%, s.d. = 5), Welch's *t*_54.30_ = 6.788, *p* < 0.001, *d* = 1.70. The experienced (*M* = 82%, s.d. = 5) and inexperienced cyclists (*M* = 83%, s.d. = 6) were in the same level of accuracy, Welch's *t*_13.96_ = −0.395, *p* = 0.70.

Accuracy was positively correlated with total points (*r* = 0.78). Children collected fewer points (*M* = 148.67, s.d. = 32.43) than adults (*M* = 191.95, s.d. = 23.42), Welch's *t*_54.30_ = 5.883, *p* < 0.001, *d* = 1.48. The difference between experienced and inexperienced adults was not significant (*M* = 194.57, s.d. = 19.43 versus *M* = 187.38, s.d. = 30.13), Welch's *t*_10.41_ = 0.607, *p* = 0.56.

In further analyses, the adults were treated as a single group. Because points correlated positively with performance measured by accuracy, we did not analyse the points but concentrated on performance.

We calculated hit rates for the locations with overt and covert targets as well as the correct rejection rate for empty locations. These measures were calculated for the game clips and three practice clips separately (table 1). It can be seen that there is no statistically significant difference between the covert and overt targets in the game. Therefore, overt and covert targets were collapsed for further analysis. The practice clips were excluded from the further analysis of performance and learning because it can be assumed that performance on them strongly reflects learning the logic of the game itself, rather than any skill related to SA.

**Table 1.** The mean percentage of correct answers (%) by the type of locations and participant groups in the game and tutorial clips. Between-subjects standard deviations are given in parentheses. For locations with overt or covert targets, a correct answer is to select the location, and for empty locations, the correct answer is to not select it.

location	children	all adults	inexp. adults	exp. adults
game				
overt	78 (9)	89 (6)	89 (7)	88 (6)
covert	78 (12)	81 (11)	75 (13)	85 (8)
no target	63 (13)	77 (13)	81 (11)	76 (15)
practice				
overt	68 (23)	80 (22)	79 (25)	81 (22)
covert	53 (24)	68 (24)	54 (25)	76 (20)
no target	54 (38)	77 (25)	62 (23)	86 (23)

### 3.1. Learning effect

The order of the video clips in the game was randomized for each participant. The learning effect can be investigated by dividing the game into three temporal phases—start, middle and ending phase—and analysing change over these phases. Each phase consisted of 10 video clips.

**Figure 2.**
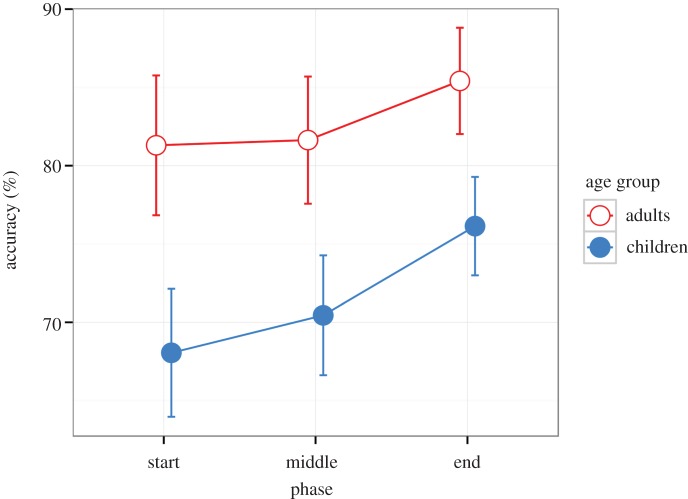
Adults' and children's accuracy during the temporal phases of the game. The figure shows means and 95% CIs.

Accuracy increased from start to end, *F*_2,112_ = 5.928, *p* = 0.004, $\eta_{p}^{2}=0.096$ (figure 2). Polynomial contrasts indicated that the increase was linear. Adults had a higher accuracy than children (*M* = 81%, 95% CI [79, 84] versus *M* = 70%, 95% CI [68, 73]), *F*_1,56_ = 40.510, *p* < 0.001, $\eta_{p}^{2}=0.420$. There was no interaction of phase and age group, *F*_2,112_ = 0.572, *p* = 0.57, indicating that the learning effect was not statistically significantly different between the adults and children. (See electronic supplementary material, figure S1 for individual changes in accuracy from the start to the end.)

Average response time was calculated for each phase. Response time decreased from the start (*M* = 4.82 s, 95% CI [4.36, 5.28]) to the end (*M* = 3.47 s, 95% CI [3.01, 3.93]), *F*_1.37, 76.84_ = 22.239, *p* < 0.001, $\eta_{p}^{2}=0.284$, degrees of freedom corrected with Greenhouse–Geisser due to sphericity (ε = 0.686). Polynomial contrasts indicated a linear decrease. The effect of age group was not significant, *F*_1,56_ = 0.484, *p* = 0.49, nor was the interaction of age group and phase, *F*_1.37, 76.84_ = 0.446, *p* = 0.57.

### 3.2. Sensitivity index *d*′ and response bias

To confirm that the improvement in accuracy is linked to increased sensitivity to the targets and not to changes in response bias, change over the phases was also investigated using SDT measures.

The raw (untransformed) hit rate and false alarm rates are shown in table 2. The general trend was that the hit rate increased and the false alarm rate decreased over the phases. In line with this, the sensitivity index *d*′ increased from the start to the end, *F*_2,112_ = 5.393, *p* = 0.006, $\eta_{p}^{2}=0.088$. Polynomial contrasts indicated that the increase was linear. Adults had a larger *d*′ (*M* = 1.78, 95% CI [1.62, 1.93]) than children (*M* = 1.04, 95% CI [0.88, 1.19]), *F*_1,56_ = 43.169, *p* < 0.001, $\eta_{p}^{2}=0.435$, and there was no interaction of age group and phase, *F*_2,112_ = 0.477, *p* = 0.62.

**Table 2.** Hit rate (%), false alarm rate (%), observed criterion, optimal criterion and response bias by the phase of game and age group. Optimal criterion is adjusted by the higher prevalence of locations with a target than without one. Bias was calculated as the difference between the observed criterion and the optimal criterion. Averages and between-subjects standard deviations in parentheses are reported.

	start	middle	end
hit rate (%)			
children	74 (11)	79 (12)	82 (10)
adults	87 (9)	86 (10)	88 (8)
false alarm rate (%)			
children	41 (18)	40 (18)	32 (18)
adults	26 (20)	24 (19)	19 (12)
*d*′			
children	0.88 (0.70)	1.06 (0.69)	1.43 (0.70)
adults	1.74 (0.76)	1.82 (0.69)	2.04 (0.64)
observed criterion			
children	0.24 (0.47)	0.28 (0.51)	0.50 (0.56)
adults	0.68 (0.61)	0.76 (0.62)	0.88 (0.46)
optimal criterion			
children	−0.20 (1.55)	0.39 (1.20)	0.45 (0.51)
adults	0.64 (0.54)	0.75 (0.42)	0.84 (0.39)
response bias			
children	0.44 (1.45)	−0.11 (1.25)	0.05 (0.40)
adults	0.03 (0.28)	0.00 (0.39)	0.04 (0.30)

Both groups used observed criteria which were close to the optimal criteria, resulting in bias close to zero (adults *M* = 0.04, 95% CI [−0.15, 0.22], children *M* = 0.14, 95% CI [−0.05, 0.33]). In bias, there was no learning effect, *F*_1.48,82.77_ = 1.324, *p* = 0.27 (ε = 0.739), no difference between age groups, *F*_1,56_ = 0.607, *p* = 0.44, nor interaction of age group and phase, *F*_1.48,82.77_ = 1.148, *p* = 0.31.

### 3.3. Eye movements

Eye movements were analysed for 10 targets from their first appearance until the moment the video was masked. These targets were distinct from other targets in the clip and unique enough so that glancing at them required moving the eyes from the direction of the movement. We first calculated the percentage of targets the participants looked toward. For the targets looked toward at least once, the average frequency of fixations, the first fixation latency (from the moment when a target was visible) and the total dwell time were calculated. For the analysis, averages of these three measures were calculated for each participant (table 3).

**Table 3.** Summary of the eye movement parameters for adults and children. Significant (*p* < 0.05) differences are denoted with an asterisk.

	children (*n* = 32)		adults (*n* = 19)	
	*M*	(s.d.)	*M*	(s.d.)
percentage of the targets looked*	35%	(16)	55%	(21)
avg. glance freq*	2.01	(0.71)	1.49	(0.39)
avg. first glance latency	2.48 s	(0.77)	1.97 s	(0.97)
avg. glance dwell time	0.74 s	(0.38)	1.16 s	(0.84)

Adults looked at a higher percentage of the targets than children, Welch's *t*_30.63_ = 3.433, *p* = 0.002, *d* = 1.06. When they looked, they looked more often, Welch's *t*_24.56_ = 2.982, *p* = 0.006, *d* = 0.99. The children were slower than adults to make the first glance to a target and spent less time looking at the examined targets than adults, but these differences were not statistically significant, Welch's *t*_31.41_ = −1.937, *p* = 0.061, *d* = 0.59, and Welch's *t*_22.46_ = 2.036, *p* = 0.054, *d* = 0.70.

Linear regressions were used to predict the accuracy based on the four eye movement parameters. The only eye movement parameter that significantly predicted accuracy was the percentage of targets looked at, *B* = 0.0068, s.e. = 0.0033, *t*_49_ = 2.04, *p* = 0.046, adj. *R*^2^ = 0.06.

### 3.4. Working memory capacity

Adults had distinctly higher PCU scores in the counting span task (*M* = 0.761, s.d. = 0.135) than children (*M* = 0.298, s.d. = 0.159), Welch's *t*_47.83_ = 11.697, *p* < 0.001, Cohen's *d* = 3.07. Linear regression models were used to investigate whether PCU scores explained accuracy in the game. Accuracy was calculated over all the game clips. Model 1 with only the PCU score as a predictor explained a significant proportion of variance in the accuracy, adj. *R*^2^ = 0.34, *F*_1,55_ = 30.24, *p* < 0.001. A higher WMC predicted a higher accuracy in the game, *B* = 1.087, s.e. = 0.198, *t*_55_ = 5.499, *p* < 0.001. However, Model 2 with the age group factor (children = 0, adults = 1) alone explained an even larger proportion of variance in the accuracy, adj. *R*^2^ = 0.43, *F*_1,55_ = 42.57, *p* < 0.001, B = 0.335, s.e. = 0.051, *t*_55_ = 6.525, *p* < 0.001. Additional analyses showed that adding the age group factor to Model 1 increased adj. *R*^2^ significantly, Δ*R*^2^ = 0.08, *F*_1, 54_ = 8.55, *p* = 0.005, but adding PCU score to Model 2 did not, Δ*R*^2^ = −0.01, *F*_1, 54_ = 0.646, *p* = 0.43.

**Figure 3.**
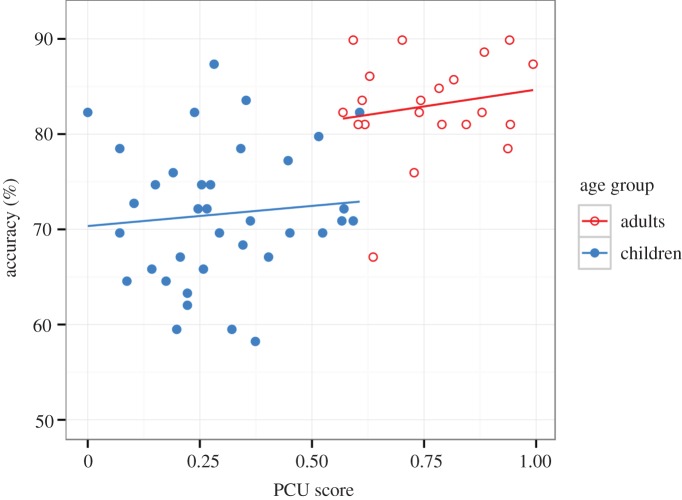
The scatter plots of PCU scores in the counting span task (on the *x*-axis) and accuracy in the game (on the *y*-axis) with regression lines. Note that the regression lines have been drawn with untransformed accuracy values for illustration. (A version with logit transformed accuracy, which shows an almost identical pattern, has been included in the electronic supplementary material, figure S2.)

These regression models suggest that variation in game performance and in PCU score can be explained by the age group factor. This is especially clear when linear regressions models were calculated for adults and children separately. PCU score did not predict accuracy among adults (adj. *R*^2^ = −0.02, *F*_1,19_ = 0.545, *p* = 0.47), nor for children (adj. *R*^2^ = −0.02, *F*_1,34_ = 0.231, *p* = 0.63) (see figure 3 for illustration).

### 3.5. Players' feedback on the game

Players were asked their opinions of the game at the end. They chose one or more adjectives to describe the game from ‘educational’, ‘fun’, ‘hard’, ‘boring’, ‘overly easy’ and ‘thrilling’. Their choices are shown in table 4 as percentages. They were also asked if they would play this kind of game again. In total, 59% of all players answered ‘yes’ (27% of adults and 78% of children), 39% answered ‘maybe’ (64% of adults and 22% of children) and 3% said ‘no’ (two adults).

**Table 4.** Percentages of players who described the game with the given adjectives.

adjective	all players (%)	adults (%)	children (%)
educational	76	55	92
fun	91	77	100
hard	41	27	50
boring	3	0	6
overly easy	5	0	8
thrilling	16	5	22

## 4. Discussion

In this study, we tested a prototype of a video-based learning game designed to train players' SA in cycling. The game was a gamified version of the SA test used by Lehtonen *et al*. [8] and was tested with 9- to 10-year-old children and adults. The first aim of the study was to investigate if the players' SA would improve while playing the game. The second aim was to find out if WMC is related to SA, especially among children who have a more limited WMC than adults.

### 4.1. Learning

Both children and adults improved in accuracy toward the end of the game, and there was no statistically significant difference between the groups in this improvement. The increase in accuracy was accompanied by a decrease in response time, which would be expected if the players learn more effective ways to acquire SA. Even though the improvement in the accuracy was small, it is noteworthy that it was produced after only playing for a short time.

In the signal detection theory-based analysis, the sensitivity index *d*′ confirmed that players indeed become better able to discriminate between locations with and without a target. This suggests that the improvement in accuracy was not due to a change in the response bias, e.g. a player who selects very few locations could increase their accuracy simply by randomly selecting more locations because in the game there were more locations with a target than without. The players used criteria which were close to the optimal criteria calculated by taking into account the higher prevalence of locations with a target than without one.

The uneven reward structure of the game could have provided a strong incentive to select locations if unsure because hits were rewarded more than false alarms were penalized. However, the players matched their criterion to the optimal criterion which was adjusted by the prevalence of targets only. If they would have adjusted for the points too, they should have used less stringent criteria. This suggests that either the participants chose to use a strategy which maximizes the accuracy, or that they did not understand the point system.

### 4.2. Adults and children

Expectedly, adults performed better at the game than children. They also looked at the targets more often than children. Other measures were in the expected direction, but they were not significant. The percentage of targets that were looked at also predicted a modest amount of variance in the accuracy. This suggests that performance in the game is related to visual scanning of the environment, which is important for acquisition of SA.

Previous studies [7,8,30] have found that children are especially poor at recognizing covert hazards. Surprisingly, there was no difference between the overt and covert targets in this study. The hit rates in the practice clips suggest that players could have been less accurate with covert targets during the practice clips, but they quickly learned to look for them as a result of explicit feedback. This raises two points to consider in the future research: First, have previous studies found a difference between overt and covert latent hazards only because they have failed to explain what a covert hazard is? Second, if the covert hazards are more challenging, is it possible to teach children to look for them by giving them clear feedback as in this study?

This study did not show the expected difference between inexperienced and experienced adult cyclists. The previous study used a comparable task, videos and participants and also did not find a difference [8]. There are multiple possible reasons for this. First, the sample size was limited. Second, it might be that the situations in the game were so simple that they could not capture the relevant aspects of SA. Regardless of their cycling experience, adults typically have a lot of experience as pedestrians and many are also frequent car drivers. It is possible they can apply the skills acquired from these roles, even though the specific hazards are different. Third, it might be that the criteria for being an experienced cyclist were not strict enough. Fourth, it is possible that typical commuting or leisure cycling does not push cyclists to develop their SA in the same way that car driving does.

In this study, children and adults performed exactly the same tasks. When comparing children and adults in cognitive tasks, it is often a problem that tasks designed for adults are too hard or uninteresting for children. From that point of view, it is noteworthy that a majority of children and adults regarded the game positively. Thus, the children's poorer performance in the game is unlikely to be due to e.g. lack of motivation. There were no floor or ceiling effects in the game or in the counting span task.

### 4.2. Working memory capacity and situation awareness

The second aim of the study was to investigate the effect of WMC on SA. Children expectedly had lower WMC than adults. A higher WMC was associated with better performance in the game when the adults and children were investigated together, but the association was non-existent when the groups were investigated separately. The age group factor explained a larger part of the variance in accuracy than PCU score. These results suggest that while 9 to 10 year olds had both a lower WMC and a poorer SA than adults, the performance in the game cannot be explained by the WMC. Instead, there must be some other factor involved.

The counting span task measures especially domain-general or executive working memory [46,55]. Recently, Jipp & Ackerman [57] found that executive working memory influences SA in a highly automated air traffic control task. They interpreted these results to mean that executive working memory counts when the mental models needed in a task are highly complex, which probably was not the case in this study. In the game, the acquisition and maintenance of SA may require mostly domain-dependent attentional skills. For example, MacKenzie & Harris [58] found that the Multiple Object Tracking task predicted driving performance among adults. For 9 to 10 year olds the number of objects correctly tracked in the Multiple Object Tracking task is still lower than for young adults [32,33]. On the other hand, it is possible the age group effect is related to experience. Adults have greater experience with traffic than children, which may help them to search for the relevant information and interpret the situation in a more task-relevant way.

The lack of significant association of WMC and game performance indicates that 9- to 10-year-old children's and adults' SA is not limited by their WMC. However, in this game they watched videos from traffic situations. In real traffic, it is necessary to control movements and plan the route simultaneously, which requires dual tasking. Consequently, children's low WMC may negatively affect the cognitive processes underlying the acquisition and maintenance of SA in real traffic [36,37,47,48]. However, for training purposes, it might be even beneficial that the game does not have dual-task components present in real cycling because those could impair learning by overloading children's WMC [59].

### 4.3. Limitations

The results suggest that the game may improve SA, but it is important to note that the current design was not able to assess if the training in the game transfers to real cycling. For example, it is possible that players can learn visual strategies which support them in the game but would negatively affect steering or balance in real cycling. In addition, the allocation of targets may have helped the players. If a clip had three locations, there was never more than two locations with a target, and if there were two locations, there was more often one location with a target than two locations with a target. It is possible that players learned to utilize this statistical feature of the stimuli for their decisions. Further experiments with a more balanced allocation of targets should be conducted to rule this out. Even if the game improved SA in real cycling, it would still be necessary to evaluate how long term the effect would be. The case for learning would have been stronger if some other SA tasks would have been used to confirm learning.

Video-based methods are limited in their visual field of view. In real cycling, the cyclists are able to turn their head to their sides, so in this case the choice of the situations is limited. However, using 360° camera technology or virtual reality these limitations could be easily solved. In this game, there was no auditory information from the environment available.

In this study, eye tracking was used as a complementary measure to investigate whether performance in the game would be linked to glances toward the targets. The small number of targets analysed makes it difficult to draw strong conclusions based on the data. It should be also noted that the viewing distance varied because it was adjusted individually so that all the participants could reach the touch screen.

### 4.4. Conclusion

The current results suggest that the game could be used to train child and adult cyclists' SA, and thus help them to anticipate and avoid hazards. More research is needed to investigate whether the improvement is permanent and how training in the game transfers to real-life cycling. Children had a lower performance in the game as well as a lower WMC. However, the analysis clearly suggested that SA is not directly related to WMC, but that some other factors are involved. Future studies are needed to establish if the difference can be explained by experience in traffic or by the level of perceptual and cognitive development.

## Supplementary Material

Supplementary Tables and Figures

## Supplementary Material

Datasets and code for the statistical analyses

## Footnotes

1 Given the fact that there were more locations with a target than locations without.

2 The auto-exposure priority was turned on, which probably overloaded the hardware.

## Data accessibility

The datasets and analysis code have been uploaded as part of the electronic supplementary material. The working memory task is available at https://github.com/kiistala/working-memory-span-tasks-for-tablet.
