# Supplementary material for: Game-based situation awareness training for child and adult cyclists
Source: R Soc Open Sci. 2017 Mar 22;4(3):160823. doi: 10.1098/rsos.160823 (PMC5383826; doi:10.1098/rsos.160823)
Supplement: Supplementary Tables and Figures [file rsos160823supp1.pdf]

Supplementary Tables and Figures

Game-based situation awareness training for child and adult cyclists
Lehtonen, Airaksinen, Kanerva, Rissanen, Ränninranta, Åberg

Supplementary Table 1. Locations and targets in the game clips. Empty, Overt and Covert columns show the number of locations without or with a specific kind of target per a clip. Number of clips displays how many video clips with this distribution of targets there were. For example, there was one clip with one overt and one covert target and without any empty locations (the first row).

Empty	Overt	Covert	Number of locations	Number of clips
0	1	1	2	1
0	2	0	2	1
1	0	1	2	2
1	1	0	2	7
1	0	2	3	1
1	1	1	3	7
1	2	0	3	5
2	1	0	3	6

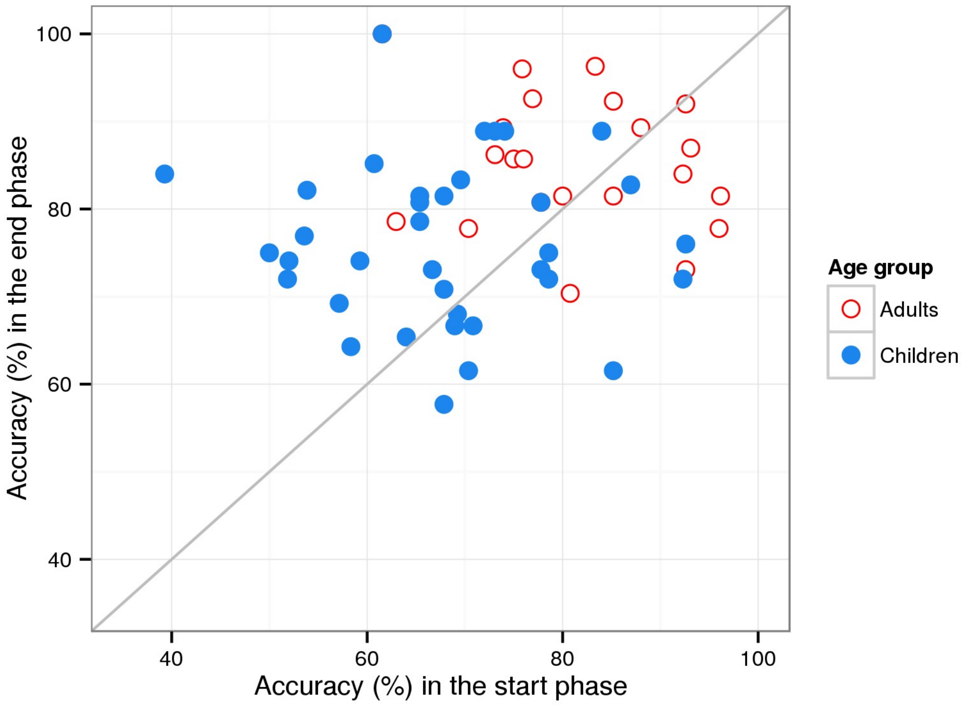

Supplementary Figure 1. Change in the accuracy from the start phase to the end phase for each player. Age group is denoted with colour and shape. Points above the abline show increase and points below decrease in accuracy.

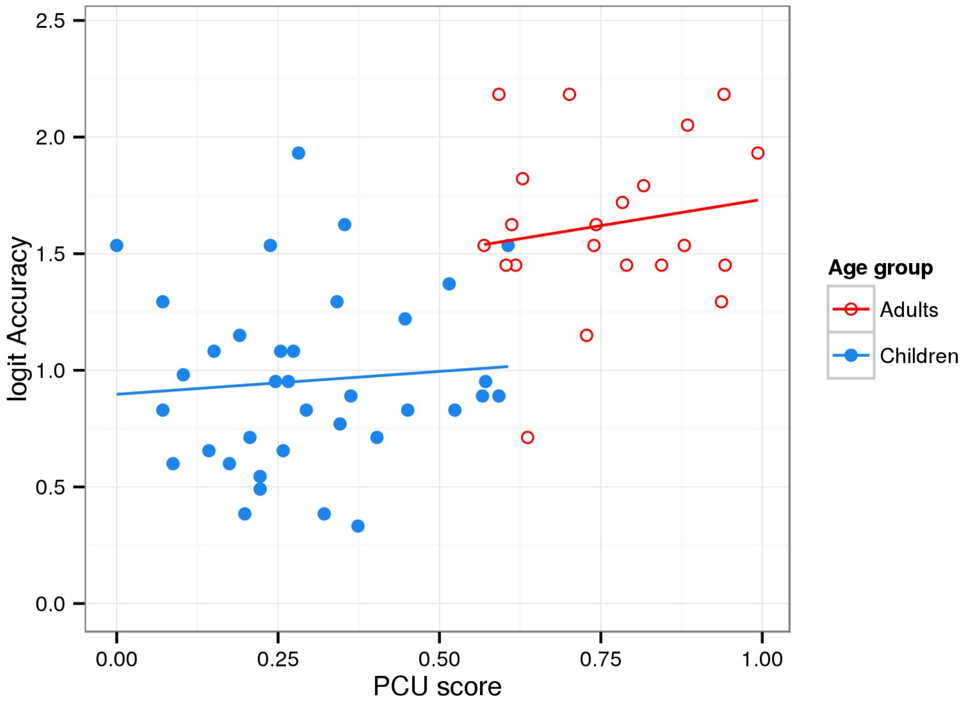

Supplementary Figure 2. The scatter plots of PCU scores in the Counting Span task (on the x-axis) and logit transformed accuracy in the game (on the y-axis) with regression lines.
