# Supplementary material for: Game-based situation awareness training for child and adult cyclists
Source: R Soc Open Sci. 2017 Mar 22;4(3):160823. doi: 10.1098/rsos.160823 (PMC5383826; doi:10.1098/rsos.160823)
Supplement: Datasets and code for the statistical analyses [file rsos160823supp2.zip › RSOS_Results_Rev2.1.html]

Results Revision 2


# Results Revision 2

#### *Author: Esko Lehtonen*

#### *30.01.2017, glance-performance plots corrected 12.03.2017*

```
# Import answer data
answers.game.final <- read.table("data/rsos_answers_game.txt", sep=" ", header=TRUE)
answers.practise.final <- read.table("data/rsos_answers_practise.txt", sep=" ", header=TRUE)
clip.times <- read.table("data/rsos_clip_times.txt", sep=" ", header=TRUE)

# Load WMC data
wmc <- read.table("data/rsos_wmc.txt", sep=" ", header=T)

# Load glances data
glances <- read.table("data/rsos_glances_to_targets.txt", sep=" ", header=TRUE)

library(plyr)
library(ggplot2)
library(ez)
library(lsmeans)
```

```
## Loading required package: estimability
## 
## Attaching package: 'lsmeans'
## 
## The following object is masked from 'package:base':
## 
##     rbind
```

```
library(knitr)
library(psych)
```

```
## 
## Attaching package: 'psych'
## 
## The following object is masked from 'package:ggplot2':
## 
##     %+%
```

```
#Create a variable set3, which divides the videos to three phases.

answers.game.final$set3 <- ordered(ceiling((answers.game.final$query_id + 1) / 10))
clip.times$set3 <- ordered(ceiling((clip.times$query_id + 1) / 10))


#Add point information to the answers
answers.game.final$points <- 0
answers.game.final$points[(answers.game.final$targetType3 != 'nothing') & 
                            (answers.game.final$answer == 'present')] <- 5
answers.game.final$points[(answers.game.final$targetType3 != 'nothing') & 
                            (answers.game.final$answer == 'notpresent')] <- -5
answers.game.final$points[(answers.game.final$targetType3 == 'nothing') & 
                            (answers.game.final$answer == 'present')] <- 0
answers.game.final$points[(answers.game.final$targetType3 == 'nothing') & 
                            (answers.game.final$answer == 'notpresent')] <- 1


# set the defaults contrast right for type III sum of squares
options(contrasts = c("contr.sum", "contr.poly"))
```

# Helper functions

```
sigdetLoglinear <- function(dframe) {
  # Use loglinear transformed values (suffix _SDT) to deal with 0 and 1 in hit and fa rates.

  dframe <- within(dframe, 
    { 
      Phit_SDT <- (hitN + 0.5) / (signalTrialN + 1)
      PfalseAlarm_SDT <- (falseAlarmN + 0.5) / (noiseTrialN +1)

      # basic SDT
      dprime <- qnorm(Phit_SDT) - qnorm(PfalseAlarm_SDT)
      criterion <- - qnorm(PfalseAlarm_SDT)

      # calculate optimal criterion by adjusting for the prevalence of signals
      s <- (signalTrialN / trialsN) # prevalence
      optimalBias <- -logit(s) # optimal bias as beta value, Wickens, 2.15 
      optimalCriterion <- 0.5*dprime + (optimalBias / dprime) # convert to criterion
      bias <- criterion - optimalCriterion 
      
      # calculate optimal criterion by adjusting for points also
      costMiss <- 5 - -5 # V(hit) - V(miss) Wickens, p 38
      costFalseAlarm <- 1 - 0 # V(correct rejection) - V(false alarm)  Wickens, p 38
      optimalBiasWithValue <- log(costFalseAlarm / costMiss) - logit(s) # Wickens, 2.15
      optimalCriterionWithValue <- 0.5*dprime + (optimalBiasWithValue / dprime)
      biasWithValue <- criterion - optimalCriterionWithValue
    })
    
  return(dframe)
}


cohen.d <- function(y, x) {
  M1 <- aggregate(y ~ x, FUN=mean)[1,2]
  SD1 <- aggregate(y ~ x, FUN=sd)[1,2]
  N1 <- aggregate(y ~ x, FUN=length)[1,2]
  M2 <- aggregate(y ~ x, FUN=mean)[2,2]
  SD2 <- aggregate(y ~ x, FUN=sd)[2,2]
  N2 <- aggregate(y ~ x, FUN=length)[2,2]
  
  dM <- abs(M1 - M2)
  SDpooled = sqrt( (N1*(SD1**2) + N2*(SD2**2)) / (N1+N2) )
  return (dM / SDpooled)
}


# Partial eta squared effect size for ANOVA tables produced by ezANOVA
pEtaSq <- function(ANOVA) {
  pes <- ANOVA$SSn / (ANOVA$SSn + ANOVA$SSd)
  ANOVA$pes <- pes
  return (ANOVA)
  }
```

# Hit and correct rejection rates by target type

For game clips

```
# Note that calculation for Phit etc. works here fine because locations with and without a target
# are separate 'target types'

tt3 <- ddply(answers.game.final, .(player_id, age_grp, experience_grp, targetType3), summarise,
          Phit = mean(hit),
          PcorrectRejection = mean(correctRejection))

desc <- ddply(tt3, .(age_grp, targetType3), summarise,
      Phit.mean = mean(100*Phit),
      Phit.sd = sd(100*Phit),
      PcorrectRejection.mean = mean(100*PcorrectRejection),
      PcorrectRejection.sd = sd(100*PcorrectRejection),
      N = length(player_id))
kable(desc, digits=0)
```

| age\_grp | targetType3 | Phit.mean | Phit.sd | PcorrectRejection.mean | PcorrectRejection.sd | N |
| --- | --- | --- | --- | --- | --- | --- |
| A | nothing | 0 | 0 | 77 | 13 | 22 |
| A | occlusion | 81 | 11 | 0 | 0 | 22 |
| A | roaduser | 89 | 6 | 0 | 0 | 22 |
| C | nothing | 0 | 0 | 63 | 13 | 36 |
| C | occlusion | 78 | 12 | 0 | 0 | 36 |
| C | roaduser | 78 | 9 | 0 | 0 | 36 |

```
desc <- ddply(tt3, .(experience_grp, targetType3), summarise,
      Phit.mean = mean(100*Phit),
      Phit.sd = sd(100*Phit),
      PcorrectRejection.mean = mean(100*PcorrectRejection),
      PcorrectRejection.sd = sd(100*PcorrectRejection),
      N = length(player_id))
kable(desc, digits=0)
```

| experience\_grp | targetType3 | Phit.mean | Phit.sd | PcorrectRejection.mean | PcorrectRejection.sd | N |
| --- | --- | --- | --- | --- | --- | --- |
| child | nothing | 0 | 0 | 63 | 13 | 36 |
| child | occlusion | 78 | 12 | 0 | 0 | 36 |
| child | roaduser | 78 | 9 | 0 | 0 | 36 |
| exp (twice per week or more) | nothing | 0 | 0 | 76 | 15 | 14 |
| exp (twice per week or more) | occlusion | 85 | 8 | 0 | 0 | 14 |
| exp (twice per week or more) | roaduser | 88 | 6 | 0 | 0 | 14 |
| inexp (twice per month or less) | nothing | 0 | 0 | 81 | 11 | 8 |
| inexp (twice per month or less) | occlusion | 75 | 13 | 0 | 0 | 8 |
| inexp (twice per month or less) | roaduser | 89 | 7 | 0 | 0 | 8 |

For practice clips

```
tt3.practise <- ddply(answers.practise.final, .(player_id, age_grp, experience_grp, targetType3), summarise,
          Phit = mean(hit),
          PcorrectRejection = mean(correctRejection))

desc <- ddply(tt3.practise, .(age_grp, targetType3), summarise,
      Phit.mean = mean(100*Phit),
      Phit.sd = sd(100*Phit),
      PcorrectRejection.mean = mean(100*PcorrectRejection),
      PcorrectRejection.sd = sd(100*PcorrectRejection),
      N = length(player_id))
kable(desc, digits=0)
```

| age\_grp | targetType3 | Phit.mean | Phit.sd | PcorrectRejection.mean | PcorrectRejection.sd | N |
| --- | --- | --- | --- | --- | --- | --- |
| A | nothing | 0 | 0 | 77 | 25 | 22 |
| A | occlusion | 68 | 24 | 0 | 0 | 22 |
| A | roaduser | 80 | 22 | 0 | 0 | 22 |
| C | nothing | 0 | 0 | 54 | 38 | 36 |
| C | occlusion | 53 | 24 | 0 | 0 | 36 |
| C | roaduser | 68 | 23 | 0 | 0 | 36 |

```
desc <- ddply(tt3.practise, .(experience_grp, targetType3), summarise,
      Phit.mean = mean(100*Phit),
      Phit.sd = sd(100*Phit),
      PcorrectRejection.mean = mean(100*PcorrectRejection),
      PcorrectRejection.sd = sd(100*PcorrectRejection),
      N = length(player_id))
kable(desc, digits=0)
```

| experience\_grp | targetType3 | Phit.mean | Phit.sd | PcorrectRejection.mean | PcorrectRejection.sd | N |
| --- | --- | --- | --- | --- | --- | --- |
| child | nothing | 0 | 0 | 54 | 38 | 36 |
| child | occlusion | 53 | 24 | 0 | 0 | 36 |
| child | roaduser | 68 | 23 | 0 | 0 | 36 |
| exp (twice per week or more) | nothing | 0 | 0 | 86 | 23 | 14 |
| exp (twice per week or more) | occlusion | 76 | 20 | 0 | 0 | 14 |
| exp (twice per week or more) | roaduser | 81 | 22 | 0 | 0 | 14 |
| inexp (twice per month or less) | nothing | 0 | 0 | 62 | 23 | 8 |
| inexp (twice per month or less) | occlusion | 54 | 25 | 0 | 0 | 8 |
| inexp (twice per month or less) | roaduser | 79 | 25 | 0 | 0 | 8 |

# Group level performance measures for the game

```
gamePerf <- ddply(answers.game.final, .(age_grp, experience_grp, player_id), summarise,
                signalTrialN = sum(targetType3 != 'nothing'),
                hitN = sum(hit),
                Phit = hitN / signalTrialN,
                Pmiss = 1 - Phit,
                
                noiseTrialN = sum(targetType3 == 'nothing'),
                falseAlarmN = sum(falseAlarm),
                PfalseAlarm = falseAlarmN / noiseTrialN,
                PcorrectRejection = 1 - PfalseAlarm,
                                
                accuracy = mean(correct),
                logitAccuracy = qlogis(accuracy), # ie.e log(accuracy/(1-accuracy)),
                trialsN = length(player_id),
                
                pointsGained = sum(points))


gameTime <- ddply(clip.times, .(age_grp, experience_grp, player_id), summarise,
                answerLatency = mean(answerLatency),
                ponderingTime = mean(ponderingTime),
                clipN = length(player_id)
                )

gamePerf <- sigdetLoglinear(gamePerf)

gamePerf <- merge(gamePerf, gameTime, by=c("player_id", "age_grp", "experience_grp"))
gamePerf.wmc <- merge(gamePerf, wmc, by="player_id", all.x=T)

gamePerf.wmc$logit_pcu_score <- qlogis(gamePerf.wmc$pcu_score)
```

# Age group differences

## Accuracy

```
describeBy(100*gamePerf$accuracy, gamePerf$age_grp)
```

```
## group: A
##    vars  n  mean   sd median trimmed  mad   min   max range  skew kurtosis
## X1    1 22 82.78 5.24  82.28   83.24 4.42 67.09 89.87 22.78 -0.93      1.4
##      se
## X1 1.12
## -------------------------------------------------------- 
## group: C
##    vars  n  mean   sd median trimmed  mad   min   max range skew kurtosis
## X1    1 36 71.61 7.25  70.89   71.58 7.51 58.23 87.34 29.11 0.15    -0.72
##      se
## X1 1.21
```

```
t.test(100*accuracy ~ age_grp, gamePerf)
```

```
## 
##  Welch Two Sample t-test
## 
## data:  100 * accuracy by age_grp
## t = 6.788, df = 54.302, p-value = 8.877e-09
## alternative hypothesis: true difference in means is not equal to 0
## 95 percent confidence interval:
##   7.874534 14.474635
## sample estimates:
## mean in group A mean in group C 
##        82.77988        71.60529
```

```
with(gamePerf, cohen.d(accuracy, age_grp))
```

```
## [1] 1.702531
```

## Points gained

```
describeBy(gamePerf$pointsGained, gamePerf$age_grp)
```

```
## group: A
##    vars  n   mean    sd median trimmed   mad min max range  skew kurtosis
## X1    1 22 191.95 23.42  199.5  193.11 20.76 143 233    90 -0.47    -0.83
##      se
## X1 4.99
## -------------------------------------------------------- 
## group: C
##    vars  n   mean    sd median trimmed   mad min max range  skew kurtosis
## X1    1 36 148.67 32.43  146.5  149.67 34.84  64 209   145 -0.28     -0.3
##     se
## X1 5.4
```

```
t.test(pointsGained ~ age_grp, gamePerf)
```

```
## 
##  Welch Two Sample t-test
## 
## data:  pointsGained by age_grp
## t = 5.8826, df = 54.302, p-value = 2.585e-07
## alternative hypothesis: true difference in means is not equal to 0
## 95 percent confidence interval:
##  28.53660 58.03916
## sample estimates:
## mean in group A mean in group C 
##        191.9545        148.6667
```

```
with(gamePerf, cohen.d(pointsGained, age_grp))
```

```
## [1] 1.475437
```

# Experience group level differences

## Accuracy

```
describeBy(100*gamePerf$accuracy, gamePerf$experience_grp)
```

```
## group: child
##    vars  n  mean   sd median trimmed  mad   min   max range skew kurtosis
## X1    1 36 71.61 7.25  70.89   71.58 7.51 58.23 87.34 29.11 0.15    -0.72
##      se
## X1 1.21
## -------------------------------------------------------- 
## group: exp (twice per week or more)
##    vars  n  mean   sd median trimmed  mad   min   max range skew kurtosis
## X1    1 14 82.43 5.23  82.28   83.09 1.88 67.09 89.87 22.78 -1.5     2.66
##     se
## X1 1.4
## -------------------------------------------------------- 
## group: inexp (twice per month or less)
##    vars n  mean   sd median trimmed  mad   min   max range skew kurtosis
## X1    1 8 83.39 5.56  82.91   83.39 7.51 75.95 89.87 13.92 0.03    -1.88
##      se
## X1 1.96
```

```
t.test(100*accuracy ~ experience_grp, gamePerf[gamePerf$age_grp == 'A',])
```

```
## 
##  Welch Two Sample t-test
## 
## data:  100 * accuracy by experience_grp
## t = -0.395, df = 13.962, p-value = 0.6988
## alternative hypothesis: true difference in means is not equal to 0
## 95 percent confidence interval:
##  -6.125903  4.220711
## sample estimates:
##    mean in group exp (twice per week or more) 
##                                      82.43348 
## mean in group inexp (twice per month or less) 
##                                      83.38608
```

```
describeBy(gamePerf$pointsGained, gamePerf$experience_grp)
```

```
## group: child
##    vars  n   mean    sd median trimmed   mad min max range  skew kurtosis
## X1    1 36 148.67 32.43  146.5  149.67 34.84  64 209   145 -0.28     -0.3
##     se
## X1 5.4
## -------------------------------------------------------- 
## group: exp (twice per week or more)
##    vars  n   mean    sd median trimmed   mad min max range  skew kurtosis
## X1    1 14 194.57 19.43    200  196.08 13.34 154 217    63 -0.85    -0.59
##      se
## X1 5.19
## -------------------------------------------------------- 
## group: inexp (twice per month or less)
##    vars n   mean    sd median trimmed   mad min max range skew kurtosis
## X1    1 8 187.38 30.13  189.5  187.38 36.32 143 233    90 0.01    -1.55
##       se
## X1 10.65
```

```
t.test(pointsGained ~ experience_grp, gamePerf[gamePerf$age_grp == 'A',])
```

```
## 
##  Welch Two Sample t-test
## 
## data:  pointsGained by experience_grp
## t = 0.6073, df = 10.405, p-value = 0.5567
## alternative hypothesis: true difference in means is not equal to 0
## 95 percent confidence interval:
##  -19.06781  33.46067
## sample estimates:
##    mean in group exp (twice per week or more) 
##                                      194.5714 
## mean in group inexp (twice per month or less) 
##                                      187.3750
```

# Points gained and accuracy

```
ggplot(gamePerf, aes(x=pointsGained, y=accuracy, colour=age_grp)) + geom_point()
```

```
with(gamePerf, cor(pointsGained, accuracy))
```

```
## [1] 0.7816375
```

```
with(gamePerf[gamePerf$age_grp == 'A',], cor(pointsGained, accuracy))
```

```
## [1] 0.2898385
```

```
with(gamePerf[gamePerf$age_grp == 'C',], cor(pointsGained, accuracy))
```

```
## [1] 0.7644237
```

# Learning over the phases

```
gamePerf.set3 <- ddply(answers.game.final, .(age_grp, experience_grp, player_id, set3), summarise,
                signalTrialN = sum(targetType3 != 'nothing'),
                hitN = sum(hit),
                Phit = hitN / signalTrialN,
                Pmiss = 1 - Phit,
                
                noiseTrialN = sum(targetType3 == 'nothing'),
                falseAlarmN = sum(falseAlarm),
                PfalseAlarm = falseAlarmN / noiseTrialN,
                PcorrectRejection = 1 - PfalseAlarm,
                                
                accuracy = mean(correct),
                logitAccuracy = qlogis(accuracy), # log(accuracy/(1-accuracy)),
                trialsN = length(player_id),
                
                pointsGained = sum(points))


gameTime.set3 <- ddply(clip.times, .(age_grp, experience_grp, player_id, set3), summarise,
                answerLatency = mean(answerLatency),
                ponderingTime = mean(ponderingTime),
                clipN = length(player_id))

gamePerf.set3 <- sigdetLoglinear(gamePerf.set3)


gamePerf.set3.locations <- ddply(answers.game.final, 
                                 .(age_grp, experience_grp, player_id, set3, locationsNum), summarise,
                signalTrialN = sum(targetType3 != 'nothing'),
                hitN = sum(hit),
                Phit = hitN / signalTrialN,
                Pmiss = 1 - Phit,
                
                noiseTrialN = sum(targetType3 == 'nothing'),
                falseAlarmN = sum(falseAlarm),
                PfalseAlarm = falseAlarmN / noiseTrialN,
                PcorrectRejection = 1 - PfalseAlarm,
                                
                accuracy = mean(correct),
                logitAccuracy = qlogis(accuracy), # log(accuracy/(1-accuracy)),
                trialsN = length(player_id),
                
                pointsGained = sum(points))
```

## Means and standard deviations

```
desc <- ddply(gamePerf.set3, .(age_grp, set3), summarise,
      Phit.mean = mean(100*Phit),
      Phit.sd = sd(100*Phit),
      PfalseAlarm.mean = mean(100*PfalseAlarm),
      PfalseAlarm.sd = sd(100*PfalseAlarm),
      N = length(player_id))
kable(desc, digits=0)
```

| age\_grp | set3 | Phit.mean | Phit.sd | PfalseAlarm.mean | PfalseAlarm.sd | N |
| --- | --- | --- | --- | --- | --- | --- |
| A | 1 | 87 | 9 | 26 | 20 | 22 |
| A | 2 | 86 | 10 | 24 | 19 | 22 |
| A | 3 | 88 | 8 | 19 | 12 | 22 |
| C | 1 | 74 | 11 | 41 | 18 | 36 |
| C | 2 | 79 | 12 | 40 | 18 | 36 |
| C | 3 | 82 | 10 | 32 | 18 | 36 |

```
desc <- ddply(gamePerf.set3, .(age_grp, set3), summarise,
      dprime.mean = mean(dprime),
      dprime.sd = sd(dprime),
      criterion.mean = mean(criterion),
      criterion.sd = sd(criterion))
kable(desc, digits=2)
```

| age\_grp | set3 | dprime.mean | dprime.sd | criterion.mean | criterion.sd |
| --- | --- | --- | --- | --- | --- |
| A | 1 | 1.74 | 0.76 | 0.68 | 0.61 |
| A | 2 | 1.82 | 0.69 | 0.76 | 0.62 |
| A | 3 | 2.04 | 0.64 | 0.88 | 0.46 |
| C | 1 | 0.88 | 0.70 | 0.24 | 0.47 |
| C | 2 | 1.06 | 0.69 | 0.28 | 0.51 |
| C | 3 | 1.43 | 0.70 | 0.50 | 0.56 |

```
desc <- ddply(gamePerf.set3, .(age_grp, set3), summarise,
      optimalCriterion.mean = mean(optimalCriterion),
      optimalCriterion.sd = sd(optimalCriterion),
      bias.mean = mean(bias),
      bias.sd = sd(bias),
      N = length(player_id))
kable(desc, digits=2)
```

| age\_grp | set3 | optimalCriterion.mean | optimalCriterion.sd | bias.mean | bias.sd | N |
| --- | --- | --- | --- | --- | --- | --- |
| A | 1 | 0.64 | 0.54 | 0.03 | 0.28 | 22 |
| A | 2 | 0.75 | 0.42 | 0.00 | 0.39 | 22 |
| A | 3 | 0.84 | 0.39 | 0.04 | 0.30 | 22 |
| C | 1 | -0.20 | 1.55 | 0.44 | 1.45 | 36 |
| C | 2 | 0.39 | 1.20 | -0.11 | 1.25 | 36 |
| C | 3 | 0.45 | 0.51 | 0.05 | 0.40 | 36 |

```
desc <- ddply(gamePerf.set3, .(age_grp, set3), summarise,
      optimalCriterionWithValue.mean = mean(optimalCriterionWithValue),
      optimalCriterionWithValue.sd = sd(optimalCriterionWithValue),
      biasWithValue.mean = mean(biasWithValue),
      biasWithValue.sd = sd(biasWithValue),
      N = length(player_id))
kable(desc, digits=2)
```

| age\_grp | set3 | optimalCriterionWithValue.mean | optimalCriterionWithValue.sd | biasWithValue.mean | biasWithValue.sd | N |
| --- | --- | --- | --- | --- | --- | --- |
| A | 1 | -1.03 | 1.51 | 1.71 | 1.06 | 22 |
| A | 2 | -0.81 | 1.37 | 1.57 | 0.96 | 22 |
| A | 3 | -0.40 | 0.79 | 1.28 | 0.55 | 22 |
| C | 1 | -4.83 | 11.46 | 5.07 | 11.39 | 36 |
| C | 2 | -1.26 | 8.93 | 1.54 | 8.97 | 36 |
| C | 3 | -1.62 | 1.60 | 2.13 | 1.26 | 36 |

## Accuracy

```
ggplot(gamePerf.set3, aes(x=set3, y=accuracy, colour=age_grp)) + geom_boxplot()
```

```
ggplot(gamePerf.set3, aes(x=set3, y=logitAccuracy, colour=age_grp)) + geom_boxplot()
```

```
## Warning in loop_apply(n, do.ply): Removed 2 rows containing non-finite
## values (stat_boxplot).
```

```
pd = position_dodge(width=0.2)
ggplot(gamePerf.set3, 
       aes(x=set3, y=100*accuracy, colour=age_grp, shape=age_grp, fill=age_grp, group=age_grp)) + 
  stat_summary(fun.data="mean_cl_normal", geom="errorbar", width=0.1, position=pd) +
  stat_summary(fun.y="mean", geom="line", position=pd) +
  stat_summary(fun.y="mean", geom="point", size=5, position=pd) +
  scale_colour_manual(name="Age group", 
                      values=c('A'='red', 'C'='dodgerblue2'), 
                      labels=c('A'='Adults', 'C'='Children')) +
  scale_shape_manual(name="Age group", 
                     values=c('A'=21, 'C'=21), 
                     labels=c('A'='Adults', 'C'='Children')) +
  scale_fill_manual(name="Age group", 
                    values=c('A'='white', 'C'='dodgerblue2'), 
                    labels=c('A'='Adults', 'C'='Children')) +
  scale_x_discrete(labels=c("Start", "Middle", "End")) +
  xlab("Phase") +
  ylab("Accuracy (%)") +
  theme_bw(base_size=10)
```

```
ggsave(file="plots/Figure2_Accuracy_and_Phase.png", dpi=300, width=14, height=10, units="cm")


fm <- ezANOVA(gamePerf.set3, dv=accuracy, wid=player_id, within=set3, between=age_grp, 
              type=3, detailed=T, return_aov=T)
```

```
## Warning: Converting "player_id" to factor for ANOVA.
```

```
## Warning: Data is unbalanced (unequal N per group). Make sure you specified
## a well-considered value for the type argument to ezANOVA().
```

```
print(fm)
```

```
## $ANOVA
##         Effect DFn DFd         SSn       SSd           F            p
## 1  (Intercept)   1  56 97.57087434 0.7153041 7638.666236 1.456764e-61
## 2      age_grp   1  56  0.51744646 0.7153041   40.510048 3.853283e-08
## 3         set3   2 112  0.11208229 1.0588938    5.927515 3.573099e-03
## 4 age_grp:set3   2 112  0.01081204 1.0588938    0.571799 5.661481e-01
##   p<.05         ges
## 1     * 0.982141059
## 2     * 0.225797024
## 3     * 0.059419750
## 4       0.006057134
## 
## $`Mauchly's Test for Sphericity`
##         Effect         W         p p<.05
## 3         set3 0.9294307 0.1336496      
## 4 age_grp:set3 0.9294307 0.1336496      
## 
## $`Sphericity Corrections`
##         Effect       GGe      p[GG] p[GG]<.05       HFe       p[HF]
## 3         set3 0.9340824 0.00442043         * 0.9651012 0.003998959
## 4 age_grp:set3 0.9340824 0.55480887           0.9651012 0.560236554
##   p[HF]<.05
## 3         *
## 4          
## 
## $aov
## 
## Call:
## aov(formula = formula(aov_formula), data = data)
## 
## Grand Mean: 0.7580867
## 
## Stratum 1: player_id
## 
## Terms:
##                   age_grp Residuals
## Sum of Squares  0.5174465 0.7153041
## Deg. of Freedom         1        56
## 
## Residual standard error: 0.113019
## Estimated effects are balanced
## 
## Stratum 2: player_id:set3
## 
## Terms:
##                      set3 age_grp:set3 Residuals
## Sum of Squares  0.1365676    0.0108120 1.0588938
## Deg. of Freedom         2            2       112
## 
## Residual standard error: 0.09723378
## Estimated effects may be unbalanced
```

```
pEtaSq(fm$ANOVA)
```

```
##         Effect DFn DFd         SSn       SSd           F            p
## 1  (Intercept)   1  56 97.57087434 0.7153041 7638.666236 1.456764e-61
## 2      age_grp   1  56  0.51744646 0.7153041   40.510048 3.853283e-08
## 3         set3   2 112  0.11208229 1.0588938    5.927515 3.573099e-03
## 4 age_grp:set3   2 112  0.01081204 1.0588938    0.571799 5.661481e-01
##   p<.05         ges        pes
## 1     * 0.982141059 0.99272223
## 2     * 0.225797024 0.41974954
## 3     * 0.059419750 0.09571698
## 4       0.006057134 0.01010749
```

```
lsmeans(fm$aov, pairwise ~ age_grp)
```

```
## NOTE: Results may be misleading due to involvement in interactions
```

```
## $lsmeans
##  age_grp    lsmean         SE df  lower.CL  upper.CL
##  A       0.8142812 0.01230289 56 0.7896356 0.8389269
##  C       0.7018923 0.01230289 56 0.6772466 0.7265379
## 
## Results are averaged over the levels of: set3 
## Confidence level used: 0.95 
## 
## $contrasts
##  contrast  estimate         SE df t.ratio p.value
##  A - C    0.1123889 0.01765802 56   6.365  <.0001
## 
## Results are averaged over the levels of: set3
```

```
lsmeans(fm$aov, poly ~ set3)
```

```
## NOTE: Results may be misleading due to involvement in interactions
```

```
## $lsmeans
##  set3    lsmean         SE    df  lower.CL  upper.CL
##  1    0.7331956 0.01374061 165.7 0.7060663 0.7603248
##  2    0.7468400 0.01374061 165.7 0.7197108 0.7739692
##  3    0.7942247 0.01374061 165.7 0.7670954 0.8213539
## 
## Results are averaged over the levels of: age_grp 
## Confidence level used: 0.95 
## 
## $contrasts
##  contrast    estimate         SE  df t.ratio p.value
##  linear    0.06102913 0.01860602 112   3.280  0.0014
##  quadratic 0.03374026 0.03222658 112   1.047  0.2974
## 
## Results are averaged over the levels of: age_grp
```

## Change in accuracy from start to end per individual

```
gamePerf.set3.wide <- reshape(gamePerf.set3, idvar="player_id", v.names=c("accuracy", "bias", "dprime"), timevar="set3", direction="wide")
```

```
## Warning in reshapeWide(data, idvar = idvar, timevar =
## timevar, varying = varying, : some constant variables
## (signalTrialN,hitN,Phit,Pmiss,noiseTrialN,falseAlarmN,PfalseAlarm,PcorrectRejection,logitAccuracy,trialsN,pointsGained,biasWithValue,optimalCriterionWithValue,optimalBiasWithValue,optimalCriterion,optimalBias,s,criterion,PfalseAlarm_SDT,Phit_SDT)
## are really varying
```

```
ggplot(gamePerf.set3.wide, 
       aes(x=100*accuracy.1, y=100*accuracy.3, colour=age_grp, fill=age_grp, shape=age_grp)) + 
  geom_point(size=3) + 
  geom_abline(colour="grey") +
  theme_bw() +
  xlim(35, 100) +
  ylim(35, 100) +
  xlab("Accuracy (%) in the start phase") +
  ylab("Accuracy (%) in the end phase") +
  scale_colour_manual(name="Age group", 
                      values=c('A'='red', 'C'='dodgerblue2'), 
                      labels=c('A'='Adults', 'C'='Children')) +
  scale_shape_manual(name="Age group", 
                     values=c('A'=21, 'C'=21), 
                     labels=c('A'='Adults', 'C'='Children')) +
  scale_fill_manual(name="Age group", 
                    values=c('A'='white', 'C'='dodgerblue2'), 
                    labels=c('A'='Adults', 'C'='Children')) + 
  theme_bw(base_size=10)
```

```
ggsave(file="plots/Supplementary_Figure_1_Change_in_Accuracy.png", dpi=300, width=14, height=10, units="cm")
```

## Points

```
# points show a similar effect to accuracy
ggplot(gamePerf.set3, aes(x=set3, y=pointsGained, colour=age_grp)) + geom_boxplot()
```

```
# there's a high correlation between points and accuracy, also when phases and groups are plotted separately
ggplot(gamePerf.set3, aes(x=pointsGained, y=accuracy, colour=age_grp)) + geom_point() + facet_grid(set3 ~ age_grp)
```

## dprime

```
ggplot(gamePerf.set3, aes(x=set3, y=dprime, colour=age_grp)) + geom_boxplot()
```

```
pd = position_dodge(width=0.2)
ggplot(gamePerf.set3, aes(x=set3, y=dprime, colour=age_grp, shape=age_grp, fill=age_grp, group=age_grp)) + 
  stat_summary(fun.data="mean_cl_normal", geom="errorbar", width=0.1, position=pd) +
  stat_summary(fun.y="mean", geom="line", position=pd) +
  stat_summary(fun.y="mean", geom="point", size=5, position=pd) +
  scale_colour_manual(name="Age group", 
                      values=c('A'='red', 'C'='dodgerblue2'), 
                      labels=c('A'='Adults', 'C'='Children')) +
  scale_shape_manual(name="Age group", 
                     values=c('A'=21, 'C'=21), 
                     labels=c('A'='Adults', 'C'='Children')) +
  scale_fill_manual(name="Age group", 
                    values=c('A'='white', 'C'='dodgerblue2'), 
                    labels=c('A'='Adults', 'C'='Children')) +
  scale_x_discrete(labels=c("Start", "Middle", "End")) +
  xlab("Phase") +
  ylab("d'") +
  theme_bw(base_size=10)
```

```
ggsave(file="plots/dprime_and_phases.png", dpi=300, width=14, height=10, units="cm")


fm <- ezANOVA(gamePerf.set3, dv=dprime, wid=player_id, within=set3, between=age_grp, 
              type=3, detailed=T, return_aov=T)
```

```
## Warning: Converting "player_id" to factor for ANOVA.
```

```
## Warning: Data is unbalanced (unequal N per group). Make sure you specified
## a well-considered value for the type argument to ezANOVA().
```

```
print(fm)
```

```
## $ANOVA
##         Effect DFn DFd         SSn      SSd           F            p p<.05
## 1  (Intercept)   1  56 366.4189808 29.15564 703.7905332 2.146717e-33     *
## 2      age_grp   1  56  22.4756120 29.15564  43.1694966 1.769892e-08     *
## 3         set3   2 112   5.0476983 52.41678   5.3927597 5.807227e-03     *
## 4 age_grp:set3   2 112   0.4464194 52.41678   0.4769367 6.219366e-01      
##           ges
## 1 0.817915208
## 2 0.216011887
## 3 0.058273971
## 4 0.005442889
## 
## $`Mauchly's Test for Sphericity`
##         Effect         W         p p<.05
## 3         set3 0.9509321 0.2506751      
## 4 age_grp:set3 0.9509321 0.2506751      
## 
## $`Sphericity Corrections`
##         Effect       GGe       p[GG] p[GG]<.05       HFe       p[HF]
## 3         set3 0.9532271 0.006624278         * 0.9859577 0.006041189
## 4 age_grp:set3 0.9532271 0.612874556           0.9859577 0.619260180
##   p[HF]<.05
## 3         *
## 4          
## 
## $aov
## 
## Call:
## aov(formula = formula(aov_formula), data = data)
## 
## Grand Mean: 1.405978
## 
## Stratum 1: player_id
## 
## Terms:
##                  age_grp Residuals
## Sum of Squares  22.47561  29.15564
## Deg. of Freedom        1        56
## 
## Residual standard error: 0.7215514
## Estimated effects are balanced
## 
## Stratum 2: player_id:set3
## 
## Terms:
##                     set3 age_grp:set3 Residuals
## Sum of Squares   6.15172      0.44642  52.41678
## Deg. of Freedom        2            2       112
## 
## Residual standard error: 0.6841104
## Estimated effects may be unbalanced
```

```
pEtaSq(fm$ANOVA)
```

```
##         Effect DFn DFd         SSn      SSd           F            p p<.05
## 1  (Intercept)   1  56 366.4189808 29.15564 703.7905332 2.146717e-33     *
## 2      age_grp   1  56  22.4756120 29.15564  43.1694966 1.769892e-08     *
## 3         set3   2 112   5.0476983 52.41678   5.3927597 5.807227e-03     *
## 4 age_grp:set3   2 112   0.4464194 52.41678   0.4769367 6.219366e-01      
##           ges         pes
## 1 0.817915208 0.926295475
## 2 0.216011887 0.435310232
## 3 0.058273971 0.087840320
## 4 0.005442889 0.008444805
```

```
lsmeans(fm$aov, pairwise ~ age_grp)
```

```
## NOTE: Results may be misleading due to involvement in interactions
```

```
## $lsmeans
##  age_grp   lsmean         SE df  lower.CL upper.CL
##  A       1.776332 0.07854583 56 1.6189855 1.933678
##  C       1.035625 0.07854583 56 0.8782783 1.192971
## 
## Results are averaged over the levels of: set3 
## Confidence level used: 0.95 
## 
## $contrasts
##  contrast  estimate        SE df t.ratio p.value
##  A - C    0.7407072 0.1127348 56    6.57  <.0001
## 
## Results are averaged over the levels of: set3
```

```
lsmeans(fm$aov, poly ~ set3)
```

```
## NOTE: Results may be misleading due to involvement in interactions
```

```
## $lsmeans
##  set3   lsmean         SE     df lower.CL upper.CL
##  1    1.223705 0.09329721 167.92 1.039518 1.407891
##  2    1.351207 0.09329721 167.92 1.167020 1.535393
##  3    1.643023 0.09329721 167.92 1.458836 1.827209
## 
## Results are averaged over the levels of: age_grp 
## Confidence level used: 0.95 
## 
## $contrasts
##  contrast   estimate        SE  df t.ratio p.value
##  linear    0.4193181 0.1309069 112   3.203  0.0018
##  quadratic 0.1643140 0.2267374 112   0.725  0.4702
## 
## Results are averaged over the levels of: age_grp
```

## Bias

```
ggplot(gamePerf.set3, aes(x=set3, y=bias, colour=age_grp)) + geom_boxplot()
```

```
pd = position_dodge(width=0.2)
ggplot(gamePerf.set3, aes(x=set3, y=bias, colour=age_grp, shape=age_grp, fill=age_grp, group=age_grp)) + 
  stat_summary(fun.data="mean_cl_normal", geom="errorbar", width=0.1, position=pd) +
  stat_summary(fun.y="mean", geom="line", position=pd) +
  stat_summary(fun.y="mean", geom="point", size=5, position=pd) +
  scale_colour_manual(name="Age group", 
                      values=c('A'='red', 'C'='dodgerblue2'), 
                      labels=c('A'='Adults', 'C'='Children')) +
  scale_shape_manual(name="Age group", 
                     values=c('A'=21, 'C'=21), 
                     labels=c('A'='Adults', 'C'='Children')) +
  scale_fill_manual(name="Age group", 
                    values=c('A'='white', 'C'='dodgerblue2'), 
                    labels=c('A'='Adults', 'C'='Children')) +
  scale_x_discrete(labels=c("Start", "Middle", "End")) +
  xlab("Phase") +
  ylab("bias") +
  ggtitle('b') +
  theme_bw(base_size=10)
```

```
ggsave(file="plots/Bias_and_phases.png", dpi=300, width=14, height=10, units="cm")


fm <- ezANOVA(gamePerf.set3, dv=bias, wid=player_id, within=set3, between=age_grp, 
              type=3, detailed=T, return_aov=T)
```

```
## Warning: Converting "player_id" to factor for ANOVA.
```

```
## Warning: Data is unbalanced (unequal N per group). Make sure you specified
## a well-considered value for the type argument to ezANOVA().
```

```
print(fm)
```

```
## $ANOVA
##         Effect DFn DFd       SSn      SSd         F         p p<.05
## 1  (Intercept)   1  56 0.9767512 41.26028 1.3256833 0.2544669      
## 2      age_grp   1  56 0.4469416 41.26028 0.6066058 0.4393475      
## 3         set3   2 112 2.3602769 99.83594 1.3239271 0.2702198      
## 4 age_grp:set3   2 112 2.0467653 99.83594 1.1480721 0.3209525      
##           ges
## 1 0.006874997
## 2 0.003157635
## 3 0.016452910
## 4 0.014298746
## 
## $`Mauchly's Test for Sphericity`
##         Effect         W            p p<.05
## 3         set3 0.6476039 6.471573e-06     *
## 4 age_grp:set3 0.6476039 6.471573e-06     *
## 
## $`Sphericity Corrections`
##         Effect       GGe     p[GG] p[GG]<.05       HFe     p[HF] p[HF]<.05
## 3         set3 0.7394283 0.2662822           0.7547057 0.2666891          
## 4 age_grp:set3 0.7394283 0.3090277           0.7547057 0.3099456          
## 
## $aov
## 
## Call:
## aov(formula = formula(aov_formula), data = data)
## 
## Grand Mean: 0.08981256
## 
## Stratum 1: player_id
## 
## Terms:
##                  age_grp Residuals
## Sum of Squares   0.44694  41.26028
## Deg. of Freedom        1        56
## 
## Residual standard error: 0.8583652
## Estimated effects are balanced
## 
## Stratum 2: player_id:set3
## 
## Terms:
##                     set3 age_grp:set3 Residuals
## Sum of Squares   3.75315      2.04677  99.83594
## Deg. of Freedom        2            2       112
## 
## Residual standard error: 0.9441357
## Estimated effects may be unbalanced
```

```
pEtaSq(fm$ANOVA)
```

```
##         Effect DFn DFd       SSn      SSd         F         p p<.05
## 1  (Intercept)   1  56 0.9767512 41.26028 1.3256833 0.2544669      
## 2      age_grp   1  56 0.4469416 41.26028 0.6066058 0.4393475      
## 3         set3   2 112 2.3602769 99.83594 1.3239271 0.2702198      
## 4 age_grp:set3   2 112 2.0467653 99.83594 1.1480721 0.3209525      
##           ges        pes
## 1 0.006874997 0.02312547
## 2 0.003157635 0.01071617
## 3 0.016452910 0.02309554
## 4 0.014298746 0.02008943
```

```
lsmeans(fm$aov, pairwise ~ age_grp)
```

```
## NOTE: Results may be misleading due to involvement in interactions
```

```
## $lsmeans
##  age_grp     lsmean         SE df    lower.CL  upper.CL
##  A       0.03758664 0.09343896 56 -0.14959408 0.2247674
##  C       0.14203849 0.09343896 56 -0.04514223 0.3292192
## 
## Results are averaged over the levels of: set3 
## Confidence level used: 0.95 
## 
## $contrasts
##  contrast   estimate        SE df t.ratio p.value
##  A - C    -0.1044519 0.1341105 56  -0.779  0.4393
## 
## Results are averaged over the levels of: set3
```

```
lsmeans(fm$aov, poly ~ set3)
```

```
## NOTE: Results may be misleading due to involvement in interactions
```

```
## $lsmeans
##  set3      lsmean        SE     df     lower.CL  upper.CL
##  1     0.24973474 0.1229399 165.89  0.007006217 0.4924633
##  2    -0.03939020 0.1229399 165.89 -0.282118727 0.2033383
##  3     0.05909315 0.1229399 165.89 -0.183635375 0.3018217
## 
## Results are averaged over the levels of: age_grp 
## Confidence level used: 0.95 
## 
## $contrasts
##  contrast    estimate        SE  df t.ratio p.value
##  linear    -0.1906416 0.1806637 112  -1.055  0.2936
##  quadratic  0.3876083 0.3129187 112   1.239  0.2181
## 
## Results are averaged over the levels of: age_grp
```

## Response time

```
# This is calcualated from gameTime.set3, because there we have the response time 
# (answerLatency) per clip

ggplot(gameTime.set3, aes(x=set3, y=answerLatency, colour=age_grp)) + geom_boxplot() + scale_y_log10()
```

```
fm <- ezANOVA(gameTime.set3, dv=answerLatency, wid=player_id, within=set3, between=age_grp, 
              type=3, detailed=T, return_aov=T)
```

```
## Warning: Converting "player_id" to factor for ANOVA.
```

```
## Warning: Data is unbalanced (unequal N per group). Make sure you specified
## a well-considered value for the type argument to ezANOVA().
```

```
print(fm)
```

```
## $ANOVA
##         Effect DFn DFd         SSn      SSd           F            p p<.05
## 1  (Intercept)   1  56 2657.722967 384.1431 387.4402551 7.798807e-27     *
## 2      age_grp   1  56    3.318320 384.1431   0.4837414 4.896088e-01      
## 3         set3   2 112   52.292171 131.6777  22.2388526 7.359885e-09     *
## 4 age_grp:set3   2 112    1.049277 131.6777   0.4462375 6.411647e-01      
##           ges
## 1 0.837462210
## 2 0.006391966
## 3 0.092045376
## 4 0.002030060
## 
## $`Mauchly's Test for Sphericity`
##         Effect         W            p p<.05
## 3         set3 0.5423839 4.936708e-08     *
## 4 age_grp:set3 0.5423839 4.936708e-08     *
## 
## $`Sphericity Corrections`
##         Effect       GGe        p[GG] p[GG]<.05       HFe        p[HF]
## 3         set3 0.6860517 9.207111e-07         * 0.6975364 7.711197e-07
## 4 age_grp:set3 0.6860517 5.676147e-01           0.6975364 5.708353e-01
##   p[HF]<.05
## 3         *
## 4          
## 
## $aov
## 
## Call:
## aov(formula = formula(aov_formula), data = data)
## 
## Grand Mean: 4.061665
## 
## Stratum 1: player_id
## 
## Terms:
##                  age_grp Residuals
## Sum of Squares    3.3183  384.1431
## Deg. of Freedom        1        56
## 
## Residual standard error: 2.619102
## Estimated effects are balanced
## 
## Stratum 2: player_id:set3
## 
## Terms:
##                      set3 age_grp:set3 Residuals
## Sum of Squares   58.71098      1.04928 131.67773
## Deg. of Freedom         2            2       112
## 
## Residual standard error: 1.084294
## Estimated effects may be unbalanced
```

```
pEtaSq(fm$ANOVA)
```

```
##         Effect DFn DFd         SSn      SSd           F            p p<.05
## 1  (Intercept)   1  56 2657.722967 384.1431 387.4402551 7.798807e-27     *
## 2      age_grp   1  56    3.318320 384.1431   0.4837414 4.896088e-01      
## 3         set3   2 112   52.292171 131.6777  22.2388526 7.359885e-09     *
## 4 age_grp:set3   2 112    1.049277 131.6777   0.4462375 6.411647e-01      
##           ges         pes
## 1 0.837462210 0.873714668
## 2 0.006391966 0.008564259
## 3 0.092045376 0.284243082
## 4 0.002030060 0.007905531
```

```
lsmeans(fm$aov, pairwise ~ age_grp)
```

```
## NOTE: Results may be misleading due to involvement in interactions
```

```
## $lsmeans
##  age_grp  lsmean        SE df lower.CL upper.CL
##  A       3.91936 0.2851073 56 3.348221 4.490498
##  C       4.20397 0.2851073 56 3.632831 4.775108
## 
## Results are averaged over the levels of: set3 
## Confidence level used: 0.95 
## 
## $contrasts
##  contrast   estimate        SE df t.ratio p.value
##  A - C    -0.2846098 0.4092072 56  -0.696  0.4896
## 
## Results are averaged over the levels of: set3
```

```
lsmeans(fm$aov, poly ~ set3)
```

```
## NOTE: Results may be misleading due to involvement in interactions
```

```
## $lsmeans
##  set3   lsmean        SE    df lower.CL upper.CL
##  1    4.821107 0.2318908 97.71 4.360910 5.281303
##  2    3.896701 0.2318908 97.71 3.436504 4.356897
##  3    3.467187 0.2318908 97.71 3.006990 3.927383
## 
## Results are averaged over the levels of: age_grp 
## Confidence level used: 0.95 
## 
## $contrasts
##  contrast   estimate        SE  df t.ratio p.value
##  linear    -1.353920 0.2074835 112  -6.525  <.0001
##  quadratic  0.494892 0.3593720 112   1.377  0.1712
## 
## Results are averaged over the levels of: age_grp
```

# Glances to preselected targets

```
# Number of available clips
length(glances$over5Hz)
```

```
## [1] 502
```

```
# Number of ok clips
sum(glances$over5Hz)
```

```
## [1] 480
```

```
glances.ok <- glances[glances$over5Hz,] 

glancePrms <- ddply(glances.ok, .(player_id, age_grp), summarise,
                    looked.pc = 100*mean(looked),
                    avgFreq = mean(freq[freq > 0]), # include only case with a glance
                    avgFirstLatency = mean(first_latency, na.rm=T),
                    avgDwellTime = mean(dwelltime, na.rm=T))

ggplot(glancePrms, aes(x=age_grp, y=looked.pc)) + geom_boxplot()
```

```
ggplot(glancePrms, aes(x=age_grp, y=avgFreq)) + geom_boxplot()
```

```
ggplot(glancePrms, aes(x=age_grp, y=avgFirstLatency)) + geom_boxplot()
```

```
ggplot(glancePrms, aes(x=age_grp, y=avgDwellTime)) + geom_boxplot()
```

```
desc <- ddply(glancePrms, .(age_grp), summarise,
                    looked.pc.M = mean(looked.pc),
                    looked.pc.SD = sd(looked.pc),
                    avgFreq.M = mean(avgFreq),
                    avgFreq.SD = sd(avgFreq))
kable(desc, digits=2)
```

| age\_grp | looked.pc.M | looked.pc.SD | avgFreq.M | avgFreq.SD |
| --- | --- | --- | --- | --- |
| A | 55.15 | 21.47 | 2.01 | 0.71 |
| C | 35.49 | 16.49 | 1.49 | 0.39 |

```
desc <- ddply(glancePrms, .(age_grp), summarise,
                    avgFirstLatency.M = mean(avgFirstLatency),
                    avgFirstLatency.SD = sd(avgFirstLatency),
                    avgDwellTime.M = mean(avgDwellTime),
                    avgDwellTime.SD = sd(avgDwellTime))
kable(desc, digits=2)
```

| age\_grp | avgFirstLatency.M | avgFirstLatency.SD | avgDwellTime.M | avgDwellTime.SD |
| --- | --- | --- | --- | --- |
| A | 1.97 | 0.97 | 1.16 | 0.84 |
| C | 2.48 | 0.77 | 0.74 | 0.38 |

```
t.test(looked.pc ~ age_grp, glancePrms)
```

```
## 
##  Welch Two Sample t-test
## 
## data:  looked.pc by age_grp
## t = 3.4334, df = 30.632, p-value = 0.00173
## alternative hypothesis: true difference in means is not equal to 0
## 95 percent confidence interval:
##   7.972722 31.332573
## sample estimates:
## mean in group A mean in group C 
##        55.14620        35.49355
```

```
with(glancePrms, cohen.d(looked.pc, age_grp))
```

```
## [1] 1.062086
```

```
t.test(avgFreq ~ age_grp, glancePrms)
```

```
## 
##  Welch Two Sample t-test
## 
## data:  avgFreq by age_grp
## t = 2.9816, df = 24.563, p-value = 0.006384
## alternative hypothesis: true difference in means is not equal to 0
## 95 percent confidence interval:
##  0.1625828 0.8910199
## sample estimates:
## mean in group A mean in group C 
##        2.012218        1.485417
```

```
with(glancePrms, cohen.d(avgFreq, age_grp))
```

```
## [1] 0.9910107
```

```
t.test(avgFirstLatency ~ age_grp, glancePrms)
```

```
## 
##  Welch Two Sample t-test
## 
## data:  avgFirstLatency by age_grp
## t = -1.9373, df = 31.406, p-value = 0.06175
## alternative hypothesis: true difference in means is not equal to 0
## 95 percent confidence interval:
##  -1.03996423  0.02645629
## sample estimates:
## mean in group A mean in group C 
##        1.971544        2.478298
```

```
with(glancePrms, cohen.d(avgFirstLatency, age_grp))
```

```
## [1] 0.5946946
```

```
t.test(avgDwellTime ~ age_grp, glancePrms)
```

```
## 
##  Welch Two Sample t-test
## 
## data:  avgDwellTime by age_grp
## t = 2.0361, df = 22.46, p-value = 0.0537
## alternative hypothesis: true difference in means is not equal to 0
## 95 percent confidence interval:
##  -0.007206648  0.838610221
## sample estimates:
## mean in group A mean in group C 
##       1.1588807       0.7431789
```

```
with(glancePrms, cohen.d(avgDwellTime, age_grp))
```

```
## [1] 0.6987798
```

## Looking and the game performance

```
GG <- merge(gamePerf.wmc, glancePrms, by=c("player_id", "age_grp"))

ggplot(GG, aes(x=looked.pc, y=accuracy, colour=age_grp, group=1)) + 
  geom_point() + stat_smooth(method="lm")
```

```
ggplot(GG, aes(x=looked.pc, y=logitAccuracy, colour=age_grp, group=1)) + 
  geom_point() + stat_smooth(method="lm")
```

```
fm <- lm(logitAccuracy ~ looked.pc, GG)
summary(fm)
```

```
## 
## Call:
## lm(formula = logitAccuracy ~ looked.pc, data = GG)
## 
## Residuals:
##      Min       1Q   Median       3Q      Max 
## -0.86157 -0.35246  0.00113  0.35725  0.99391 
## 
## Coefficients:
##             Estimate Std. Error t value Pr(>|t|)    
## (Intercept) 0.906454   0.157618   5.751 5.65e-07 ***
## looked.pc   0.006791   0.003322   2.044   0.0463 *  
## ---
## Signif. codes:  0 '***' 0.001 '**' 0.01 '*' 0.05 '.' 0.1 ' ' 1
## 
## Residual standard error: 0.4852 on 49 degrees of freedom
## Multiple R-squared:  0.07858,    Adjusted R-squared:  0.05978 
## F-statistic: 4.179 on 1 and 49 DF,  p-value: 0.04632
```

```
ggplot(GG, aes(x=avgFreq, y=logitAccuracy, colour=age_grp, group=1)) + 
  geom_point() + stat_smooth(method="lm")
```

```
fm <- lm(logitAccuracy ~ avgFreq, GG)
summary(fm)
```

```
## 
## Call:
## lm(formula = logitAccuracy ~ avgFreq, data = GG)
## 
## Residuals:
##      Min       1Q   Median       3Q      Max 
## -0.93040 -0.35034 -0.02679  0.31206  1.08491 
## 
## Coefficients:
##             Estimate Std. Error t value Pr(>|t|)    
## (Intercept)   0.8520     0.2113   4.033 0.000192 ***
## avgFreq       0.2053     0.1188   1.728 0.090313 .  
## ---
## Signif. codes:  0 '***' 0.001 '**' 0.01 '*' 0.05 '.' 0.1 ' ' 1
## 
## Residual standard error: 0.4907 on 49 degrees of freedom
## Multiple R-squared:  0.05743,    Adjusted R-squared:  0.03819 
## F-statistic: 2.985 on 1 and 49 DF,  p-value: 0.09031
```

```
ggplot(GG, aes(x=avgFirstLatency, y=logitAccuracy, colour=age_grp, group=1)) + 
  geom_point() + stat_smooth(method="lm")
```

```
fm <- lm(logitAccuracy ~ avgFirstLatency, GG)
summary(fm)
```

```
## 
## Call:
## lm(formula = logitAccuracy ~ avgFirstLatency, data = GG)
## 
## Residuals:
##      Min       1Q   Median       3Q      Max 
## -0.79776 -0.34575 -0.03751  0.33648  1.07724 
## 
## Coefficients:
##                 Estimate Std. Error t value Pr(>|t|)    
## (Intercept)      1.40639    0.19679   7.147 3.93e-09 ***
## avgFirstLatency -0.09137    0.08035  -1.137    0.261    
## ---
## Signif. codes:  0 '***' 0.001 '**' 0.01 '*' 0.05 '.' 0.1 ' ' 1
## 
## Residual standard error: 0.4989 on 49 degrees of freedom
## Multiple R-squared:  0.02571,    Adjusted R-squared:  0.005826 
## F-statistic: 1.293 on 1 and 49 DF,  p-value: 0.261
```

```
ggplot(GG, aes(x=avgDwellTime, y=logitAccuracy, colour=age_grp, group=1)) + 
  geom_point() + stat_smooth(method="lm")
```

```
fm <- lm(logitAccuracy ~ avgDwellTime, GG)
summary(fm)
```

```
## 
## Call:
## lm(formula = logitAccuracy ~ avgDwellTime, data = GG)
## 
## Residuals:
##      Min       1Q   Median       3Q      Max 
## -0.89192 -0.36614  0.01735  0.39701  1.01203 
## 
## Coefficients:
##              Estimate Std. Error t value Pr(>|t|)    
## (Intercept)    1.0339     0.1221   8.467 3.72e-11 ***
## avgDwellTime   0.1818     0.1122   1.621    0.112    
## ---
## Signif. codes:  0 '***' 0.001 '**' 0.01 '*' 0.05 '.' 0.1 ' ' 1
## 
## Residual standard error: 0.4924 on 49 degrees of freedom
## Multiple R-squared:  0.05087,    Adjusted R-squared:  0.0315 
## F-statistic: 2.626 on 1 and 49 DF,  p-value: 0.1115
```

```
fm <- lm(logitAccuracy ~ looked.pc + avgFreq + avgFirstLatency + avgDwellTime, GG)
summary(fm)
```

```
## 
## Call:
## lm(formula = logitAccuracy ~ looked.pc + avgFreq + avgFirstLatency + 
##     avgDwellTime, data = GG)
## 
## Residuals:
##      Min       1Q   Median       3Q      Max 
## -0.82301 -0.29190 -0.00037  0.33222  1.00488 
## 
## Coefficients:
##                  Estimate Std. Error t value Pr(>|t|)  
## (Intercept)      0.654339   0.451619   1.449   0.1542  
## looked.pc        0.007278   0.003301   2.205   0.0325 *
## avgFreq          0.137919   0.238280   0.579   0.5655  
## avgFirstLatency -0.028162   0.092179  -0.306   0.7614  
## avgDwellTime     0.071025   0.208138   0.341   0.7345  
## ---
## Signif. codes:  0 '***' 0.001 '**' 0.01 '*' 0.05 '.' 0.1 ' ' 1
## 
## Residual standard error: 0.4808 on 46 degrees of freedom
## Multiple R-squared:  0.1506, Adjusted R-squared:  0.07675 
## F-statistic: 2.039 on 4 and 46 DF,  p-value: 0.1045
```

# Working memory capacity

## Group level differences

No significant difference between adult groups

```
t.test(pcu_score ~ experience_grp, data=gamePerf.wmc[gamePerf.wmc$age_grp == 'A',])
```

```
## 
##  Welch Two Sample t-test
## 
## data:  pcu_score by experience_grp
## t = -1.3378, df = 15.978, p-value = 0.1997
## alternative hypothesis: true difference in means is not equal to 0
## 95 percent confidence interval:
##  -0.19164974  0.04335851
## sample estimates:
##    mean in group exp (twice per week or more) 
##                                     0.7363014 
## mean in group inexp (twice per month or less) 
##                                     0.8104470
```

Adults and children do have a significant difference in WMC

```
t.test(pcu_score ~ age_grp, data=gamePerf.wmc)
```

```
## 
##  Welch Two Sample t-test
## 
## data:  pcu_score by age_grp
## t = 11.6967, df = 47.828, p-value = 1.238e-15
## alternative hypothesis: true difference in means is not equal to 0
## 95 percent confidence interval:
##  0.3834413 0.5426484
## sample estimates:
## mean in group A mean in group C 
##       0.7610166       0.2979718
```

```
res <- ddply(gamePerf.wmc, .(age_grp), summarise,
      M = mean(pcu_score, na.rm=T),
      SD = sd(pcu_score, na.rm=T),
      N = sum(is.finite(pcu_score)))
res
```

```
##   age_grp         M        SD  N
## 1       A 0.7610166 0.1345289 21
## 2       C 0.2979718 0.1593522 36
```

```
# effect size
with(gamePerf.wmc, cohen.d(pcu_score, age_grp))
```

```
## [1] 3.072967
```

## Predicting accuracy with PCU score

Using logit transformed accuracy

```
ggplot(gamePerf.wmc, aes(x=pcu_score, y=logitAccuracy, colour=age_grp, shape=age_grp, fill=age_grp)) +
  geom_point() + 
  geom_smooth(method="lm", se=FALSE) +
  scale_colour_manual(name="Age group", 
                      values=c('A'='red', 'C'='dodgerblue2'), 
                      labels=c('A'='Adults', 'C'='Children')) +
  scale_shape_manual(name="Age group", 
                     values=c('A'=21, 'C'=21), 
                     labels=c('A'='Adults', 'C'='Children')) +
  scale_fill_manual(name="Age group", 
                    values=c('A'='white', 'C'='dodgerblue2'), 
                    labels=c('A'='Adults', 'C'='Children')) +
  ylim(qlogis(0.5), qlogis(0.92)) +
  ylab("logit Accuracy") +
  xlab("PCU score") +
  theme_bw(base_size=10)
```

```
## Warning in loop_apply(n, do.ply): Removed 1 rows containing missing values
## (stat_smooth).
```

```
## Warning in loop_apply(n, do.ply): Removed 1 rows containing missing values
## (geom_point).
```

```
ggsave(file="plots/Supplementary_Figure_2_logitAccuracy_and_PCU_score.png", dpi=300, width=14, height=10, units="cm")
```

```
## Warning in loop_apply(n, do.ply): Removed 1 rows containing missing values
## (stat_smooth).
```

```
## Warning in loop_apply(n, do.ply): Removed 1 rows containing missing values
## (geom_point).
```

```
fm1 <- lm(logitAccuracy ~ pcu_score, data=gamePerf.wmc[is.finite(gamePerf.wmc$pcu_score),])
summary(fm1)
```

```
## 
## Call:
## lm(formula = logitAccuracy ~ pcu_score, data = gamePerf.wmc[is.finite(gamePerf.wmc$pcu_score), 
##     ])
## 
## Residuals:
##      Min       1Q   Median       3Q      Max 
## -0.76720 -0.31799  0.03884  0.22368  0.93225 
## 
## Coefficients:
##             Estimate Std. Error t value Pr(>|t|)    
## (Intercept)   0.6931     0.1067   6.496 2.52e-08 ***
## pcu_score     1.0869     0.1977   5.499 1.02e-06 ***
## ---
## Signif. codes:  0 '***' 0.001 '**' 0.01 '*' 0.05 '.' 0.1 ' ' 1
## 
## Residual standard error: 0.4 on 55 degrees of freedom
## Multiple R-squared:  0.3547, Adjusted R-squared:  0.343 
## F-statistic: 30.24 on 1 and 55 DF,  p-value: 1.023e-06
```

```
fm2 <- lm(logitAccuracy ~ age_grp, data=gamePerf.wmc[is.finite(gamePerf.wmc$pcu_score),])
summary(fm2)
```

```
## 
## Call:
## lm(formula = logitAccuracy ~ age_grp, data = gamePerf.wmc[is.finite(gamePerf.wmc$pcu_score), 
##     ])
## 
## Residuals:
##      Min       1Q   Median       3Q      Max 
## -0.91310 -0.18547 -0.06572  0.19631  0.97594 
## 
## Coefficients:
##             Estimate Std. Error t value Pr(>|t|)    
## (Intercept)  1.29044    0.05132  25.144  < 2e-16 ***
## age_grp1     0.33486    0.05132   6.525 2.26e-08 ***
## ---
## Signif. codes:  0 '***' 0.001 '**' 0.01 '*' 0.05 '.' 0.1 ' ' 1
## 
## Residual standard error: 0.3738 on 55 degrees of freedom
## Multiple R-squared:  0.4363, Adjusted R-squared:  0.4261 
## F-statistic: 42.57 on 1 and 55 DF,  p-value: 2.261e-08
```

```
fm3 <- lm(logitAccuracy ~ pcu_score + age_grp, data=gamePerf.wmc[is.finite(gamePerf.wmc$pcu_score),])
summary(fm3)
```

```
## 
## Call:
## lm(formula = logitAccuracy ~ pcu_score + age_grp, data = gamePerf.wmc[is.finite(gamePerf.wmc$pcu_score), 
##     ])
## 
## Residuals:
##      Min       1Q   Median       3Q      Max 
## -0.87955 -0.21869 -0.07726  0.22481  0.98032 
## 
## Coefficients:
##             Estimate Std. Error t value Pr(>|t|)    
## (Intercept)  1.14774    0.18488   6.208 7.89e-08 ***
## pcu_score    0.26949    0.33534   0.804  0.42513    
## age_grp1     0.27247    0.09316   2.925  0.00503 ** 
## ---
## Signif. codes:  0 '***' 0.001 '**' 0.01 '*' 0.05 '.' 0.1 ' ' 1
## 
## Residual standard error: 0.375 on 54 degrees of freedom
## Multiple R-squared:  0.443,  Adjusted R-squared:  0.4223 
## F-statistic: 21.47 on 2 and 54 DF,  p-value: 1.377e-07
```

```
anova(fm1, fm3)
```

```
## Analysis of Variance Table
## 
## Model 1: logitAccuracy ~ pcu_score
## Model 2: logitAccuracy ~ pcu_score + age_grp
##   Res.Df    RSS Df Sum of Sq      F   Pr(>F)   
## 1     55 8.7981                                
## 2     54 7.5950  1    1.2031 8.5538 0.005032 **
## ---
## Signif. codes:  0 '***' 0.001 '**' 0.01 '*' 0.05 '.' 0.1 ' ' 1
```

```
anova(fm2, fm3)
```

```
## Analysis of Variance Table
## 
## Model 1: logitAccuracy ~ age_grp
## Model 2: logitAccuracy ~ pcu_score + age_grp
##   Res.Df    RSS Df Sum of Sq      F Pr(>F)
## 1     55 7.6858                           
## 2     54 7.5950  1  0.090833 0.6458 0.4251
```

```
fm.a <- lm(logitAccuracy ~ pcu_score, data=gamePerf.wmc[gamePerf.wmc$age_grp == 'A',])
summary(fm.a)
```

```
## 
## Call:
## lm(formula = logitAccuracy ~ pcu_score, data = gamePerf.wmc[gamePerf.wmc$age_grp == 
##     "A", ])
## 
## Residuals:
##      Min       1Q   Median       3Q      Max 
## -0.85707 -0.18742 -0.00363  0.20174  0.63397 
## 
## Coefficients:
##             Estimate Std. Error t value Pr(>|t|)  
## (Intercept)   1.2828     0.4709   2.724   0.0135 *
## pcu_score     0.4500     0.6098   0.738   0.4695  
## ---
## Signif. codes:  0 '***' 0.001 '**' 0.01 '*' 0.05 '.' 0.1 ' ' 1
## 
## Residual standard error: 0.3669 on 19 degrees of freedom
##   (1 observation deleted due to missingness)
## Multiple R-squared:  0.02787,    Adjusted R-squared:  -0.0233 
## F-statistic: 0.5447 on 1 and 19 DF,  p-value: 0.4695
```

```
fm.c <- lm(logitAccuracy ~ pcu_score, data=gamePerf.wmc[gamePerf.wmc$age_grp == 'C',])
summary(fm.c)
```

```
## 
## Call:
## lm(formula = logitAccuracy ~ pcu_score, data = gamePerf.wmc[gamePerf.wmc$age_grp == 
##     "C", ])
## 
## Residuals:
##      Min       1Q   Median       3Q      Max 
## -0.63831 -0.26544 -0.08016  0.22048  0.97912 
## 
## Coefficients:
##             Estimate Std. Error t value Pr(>|t|)    
## (Intercept)   0.8972     0.1373   6.534 1.76e-07 ***
## pcu_score     0.1960     0.4076   0.481    0.634    
## ---
## Signif. codes:  0 '***' 0.001 '**' 0.01 '*' 0.05 '.' 0.1 ' ' 1
## 
## Residual standard error: 0.3843 on 34 degrees of freedom
## Multiple R-squared:  0.006751,   Adjusted R-squared:  -0.02246 
## F-statistic: 0.2311 on 1 and 34 DF,  p-value: 0.6338
```

With untransformed accuracy

```
ggplot(gamePerf.wmc, aes(x=pcu_score, y=100*accuracy, colour=age_grp, shape=age_grp, fill=age_grp)) +
  geom_point() + 
  geom_smooth(method="lm", se=FALSE) +
  scale_colour_manual(name="Age group", 
                      values=c('A'='red', 'C'='dodgerblue2'), 
                      labels=c('A'='Adults', 'C'='Children')) +
  scale_shape_manual(name="Age group", 
                     values=c('A'=21, 'C'=21), 
                     labels=c('A'='Adults', 'C'='Children')) +
  scale_fill_manual(name="Age group", 
                    values=c('A'='white', 'C'='dodgerblue2'), 
                    labels=c('A'='Adults', 'C'='Children')) +
  ylim(50, 92) +
  ylab("Accuracy (%)") +
  xlab("PCU score") +
  theme_bw()
```

```
## Warning in loop_apply(n, do.ply): Removed 1 rows containing missing values
## (stat_smooth).
```

```
## Warning in loop_apply(n, do.ply): Removed 1 rows containing missing values
## (geom_point).
```

```
ggsave(file="plots/Figure3_Accuracy_and_PCU_score.png", dpi=300, width=14, height=10, units="cm")
```

```
## Warning in loop_apply(n, do.ply): Removed 1 rows containing missing values
## (stat_smooth).
```

```
## Warning in loop_apply(n, do.ply): Removed 1 rows containing missing values
## (geom_point).
```

Regression should not be done with raw accuracy, but let’s perform it for comparison.

```
fm1 <- lm(100*accuracy ~ pcu_score, data=gamePerf.wmc[is.finite(gamePerf.wmc$pcu_score),])
summary(fm1)
```

```
## 
## Call:
## lm(formula = 100 * accuracy ~ pcu_score, data = gamePerf.wmc[is.finite(gamePerf.wmc$pcu_score), 
##     ])
## 
## Residuals:
##     Min      1Q  Median      3Q     Max 
## -15.806  -5.560   1.214   4.609  15.201 
## 
## Coefficients:
##             Estimate Std. Error t value Pr(>|t|)    
## (Intercept)   67.077      1.869  35.898  < 2e-16 ***
## pcu_score     18.611      3.462   5.376  1.6e-06 ***
## ---
## Signif. codes:  0 '***' 0.001 '**' 0.01 '*' 0.05 '.' 0.1 ' ' 1
## 
## Residual standard error: 7.004 on 55 degrees of freedom
## Multiple R-squared:  0.3445, Adjusted R-squared:  0.3326 
## F-statistic: 28.91 on 1 and 55 DF,  p-value: 1.597e-06
```

```
fm2 <- lm(100*accuracy ~ age_grp, data=gamePerf.wmc[is.finite(gamePerf.wmc$pcu_score),])
summary(fm2)
```

```
## 
## Call:
## lm(formula = 100 * accuracy ~ age_grp, data = gamePerf.wmc[is.finite(gamePerf.wmc$pcu_score), 
##     ])
## 
## Residuals:
##      Min       1Q   Median       3Q      Max 
## -15.8960  -3.2509  -0.7061   4.3441  15.7365 
## 
## Coefficients:
##             Estimate Std. Error t value Pr(>|t|)    
## (Intercept)  77.2949     0.9068  85.242  < 2e-16 ***
## age_grp1      5.6896     0.9068   6.275 5.77e-08 ***
## ---
## Signif. codes:  0 '***' 0.001 '**' 0.01 '*' 0.05 '.' 0.1 ' ' 1
## 
## Residual standard error: 6.605 on 55 degrees of freedom
## Multiple R-squared:  0.4172, Adjusted R-squared:  0.4066 
## F-statistic: 39.37 on 1 and 55 DF,  p-value: 5.773e-08
```

```
fm3 <- lm(100*accuracy ~ pcu_score + age_grp, data=gamePerf.wmc[is.finite(gamePerf.wmc$pcu_score),])
summary(fm3)
```

```
## 
## Call:
## lm(formula = 100 * accuracy ~ pcu_score + age_grp, data = gamePerf.wmc[is.finite(gamePerf.wmc$pcu_score), 
##     ])
## 
## Residuals:
##      Min       1Q   Median       3Q      Max 
## -15.2671  -3.4936  -0.8346   3.8216  15.8184 
## 
## Coefficients:
##             Estimate Std. Error t value Pr(>|t|)    
## (Intercept)   74.621      3.264  22.862  < 2e-16 ***
## pcu_score      5.051      5.920   0.853  0.39736    
## age_grp1       4.520      1.645   2.748  0.00813 ** 
## ---
## Signif. codes:  0 '***' 0.001 '**' 0.01 '*' 0.05 '.' 0.1 ' ' 1
## 
## Residual standard error: 6.621 on 54 degrees of freedom
## Multiple R-squared:  0.4249, Adjusted R-squared:  0.4036 
## F-statistic: 19.95 on 2 and 54 DF,  p-value: 3.252e-07
```

```
anova(fm1, fm3)
```

```
## Analysis of Variance Table
## 
## Model 1: 100 * accuracy ~ pcu_score
## Model 2: 100 * accuracy ~ pcu_score + age_grp
##   Res.Df    RSS Df Sum of Sq      F   Pr(>F)   
## 1     55 2698.4                                
## 2     54 2367.3  1    331.13 7.5534 0.008127 **
## ---
## Signif. codes:  0 '***' 0.001 '**' 0.01 '*' 0.05 '.' 0.1 ' ' 1
```

```
anova(fm2, fm3)
```

```
## Analysis of Variance Table
## 
## Model 1: 100 * accuracy ~ age_grp
## Model 2: 100 * accuracy ~ pcu_score + age_grp
##   Res.Df    RSS Df Sum of Sq      F Pr(>F)
## 1     55 2399.2                           
## 2     54 2367.3  1    31.906 0.7278 0.3974
```

```
fm.a <- lm(100*accuracy ~ pcu_score, data=gamePerf.wmc[gamePerf.wmc$age_grp == 'A',])
summary(fm.a)
```

```
## 
## Call:
## lm(formula = 100 * accuracy ~ pcu_score, data = gamePerf.wmc[gamePerf.wmc$age_grp == 
##     "A", ])
## 
## Residuals:
##     Min      1Q  Median      3Q     Max 
## -15.015  -2.175   0.651   2.715   8.084 
## 
## Coefficients:
##             Estimate Std. Error t value Pr(>|t|)    
## (Intercept)   77.602      6.838  11.348 6.61e-10 ***
## pcu_score      7.073      8.855   0.799    0.434    
## ---
## Signif. codes:  0 '***' 0.001 '**' 0.01 '*' 0.05 '.' 0.1 ' ' 1
## 
## Residual standard error: 5.327 on 19 degrees of freedom
##   (1 observation deleted due to missingness)
## Multiple R-squared:  0.03249,    Adjusted R-squared:  -0.01843 
## F-statistic: 0.6381 on 1 and 19 DF,  p-value: 0.4343
```

```
fm.c <- lm(100*accuracy ~ pcu_score, data=gamePerf.wmc[gamePerf.wmc$age_grp == 'C',])
summary(fm.c)
```

```
## 
## Call:
## lm(formula = 100 * accuracy ~ pcu_score, data = gamePerf.wmc[gamePerf.wmc$age_grp == 
##     "C", ])
## 
## Residuals:
##     Min      1Q  Median      3Q     Max 
## -13.698  -5.003  -1.010   4.844  15.805 
## 
## Coefficients:
##             Estimate Std. Error t value Pr(>|t|)    
## (Intercept)   70.346      2.619  26.864   <2e-16 ***
## pcu_score      4.227      7.773   0.544     0.59    
## ---
## Signif. codes:  0 '***' 0.001 '**' 0.01 '*' 0.05 '.' 0.1 ' ' 1
## 
## Residual standard error: 7.328 on 34 degrees of freedom
## Multiple R-squared:  0.008622,   Adjusted R-squared:  -0.02054 
## F-statistic: 0.2957 on 1 and 34 DF,  p-value: 0.5901
```

## Predicting dprime with PCU score

In the current data dprime is highly correlated to the accuracy so the outcome is the same.

```
ggplot(gamePerf.wmc, aes(x=pcu_score, y=dprime, colour=age_grp, shape=age_grp, fill=age_grp)) + 
  geom_point() + 
  geom_smooth(method="lm", se=FALSE) +
  scale_colour_manual(name="Age group", 
                      values=c('A'='red', 'C'='dodgerblue2'), 
                      labels=c('A'='Adults', 'C'='Children')) +
  scale_shape_manual(name="Age group", 
                     values=c('A'=21, 'C'=21), 
                     labels=c('A'='Adults', 'C'='Children')) +
  scale_fill_manual(name="Age group", 
                    values=c('A'='white', 'C'='dodgerblue2'), 
                    labels=c('A'='Adults', 'C'='Children')) +
  ylab("d'") +
  xlab("PCU score") +
  theme_bw()
```

```
## Warning in loop_apply(n, do.ply): Removed 1 rows containing missing values
## (stat_smooth).
```

```
## Warning in loop_apply(n, do.ply): Removed 1 rows containing missing values
## (geom_point).
```

```
ggsave(file="plots/dprime_and_PCU_score.png", dpi=300, width=14, height=10, units="cm")
```

```
## Warning in loop_apply(n, do.ply): Removed 1 rows containing missing values
## (stat_smooth).
```

```
## Warning in loop_apply(n, do.ply): Removed 1 rows containing missing values
## (geom_point).
```

```
fm1 <- lm(dprime ~ pcu_score, data=gamePerf.wmc[is.finite(gamePerf.wmc$pcu_score),])
summary(fm1)
```

```
## 
## Call:
## lm(formula = dprime ~ pcu_score, data = gamePerf.wmc[is.finite(gamePerf.wmc$pcu_score), 
##     ])
## 
## Residuals:
##      Min       1Q   Median       3Q      Max 
## -0.89465 -0.35896  0.02873  0.27377  1.01483 
## 
## Coefficients:
##             Estimate Std. Error t value Pr(>|t|)    
## (Intercept)   0.8090     0.1219   6.636 1.49e-08 ***
## pcu_score     1.2763     0.2259   5.651 5.86e-07 ***
## ---
## Signif. codes:  0 '***' 0.001 '**' 0.01 '*' 0.05 '.' 0.1 ' ' 1
## 
## Residual standard error: 0.457 on 55 degrees of freedom
## Multiple R-squared:  0.3673, Adjusted R-squared:  0.3558 
## F-statistic: 31.93 on 1 and 55 DF,  p-value: 5.86e-07
```

```
fm2 <- lm(dprime ~ age_grp, data=gamePerf.wmc)
summary(fm2)
```

```
## 
## Call:
## lm(formula = dprime ~ age_grp, data = gamePerf.wmc)
## 
## Residuals:
##      Min       1Q   Median       3Q      Max 
## -0.90016 -0.27575 -0.07646  0.28661  1.06596 
## 
## Coefficients:
##             Estimate Std. Error t value Pr(>|t|)    
## (Intercept)  1.50283    0.05738  26.190  < 2e-16 ***
## age_grp1     0.38536    0.05738   6.716 1.02e-08 ***
## ---
## Signif. codes:  0 '***' 0.001 '**' 0.01 '*' 0.05 '.' 0.1 ' ' 1
## 
## Residual standard error: 0.4241 on 56 degrees of freedom
## Multiple R-squared:  0.4461, Adjusted R-squared:  0.4362 
## F-statistic:  45.1 on 1 and 56 DF,  p-value: 1.02e-08
```

```
fm3 <- lm(dprime ~ pcu_score + age_grp, data=gamePerf.wmc)
summary(fm3)
```

```
## 
## Call:
## lm(formula = dprime ~ pcu_score + age_grp, data = gamePerf.wmc)
## 
## Residuals:
##      Min       1Q   Median       3Q      Max 
## -0.87570 -0.27200 -0.08631  0.27679  1.07113 
## 
## Coefficients:
##             Estimate Std. Error t value Pr(>|t|)    
## (Intercept)   1.3416     0.2104   6.376 4.22e-08 ***
## pcu_score     0.3188     0.3816   0.835  0.40719    
## age_grp1      0.3192     0.1060   3.010  0.00396 ** 
## ---
## Signif. codes:  0 '***' 0.001 '**' 0.01 '*' 0.05 '.' 0.1 ' ' 1
## 
## Residual standard error: 0.4268 on 54 degrees of freedom
##   (1 observation deleted due to missingness)
## Multiple R-squared:  0.4582, Adjusted R-squared:  0.4382 
## F-statistic: 22.84 on 2 and 54 DF,  p-value: 6.506e-08
```

```
anova(fm1, fm3)
```

```
## Analysis of Variance Table
## 
## Model 1: dprime ~ pcu_score
## Model 2: dprime ~ pcu_score + age_grp
##   Res.Df     RSS Df Sum of Sq      F   Pr(>F)   
## 1     55 11.4881                                
## 2     54  9.8373  1    1.6508 9.0616 0.003963 **
## ---
## Signif. codes:  0 '***' 0.001 '**' 0.01 '*' 0.05 '.' 0.1 ' ' 1
```

```
fm.a <- lm(dprime ~ pcu_score, data=gamePerf.wmc[gamePerf.wmc$age_grp == 'A',])
summary(fm.a)
```

```
## 
## Call:
## lm(formula = dprime ~ pcu_score, data = gamePerf.wmc[gamePerf.wmc$age_grp == 
##     "A", ])
## 
## Residuals:
##      Min       1Q   Median       3Q      Max 
## -0.85255 -0.16709 -0.02093  0.23427  0.66640 
## 
## Coefficients:
##             Estimate Std. Error t value Pr(>|t|)   
## (Intercept)   1.5193     0.4889   3.108   0.0058 **
## pcu_score     0.5047     0.6331   0.797   0.4351   
## ---
## Signif. codes:  0 '***' 0.001 '**' 0.01 '*' 0.05 '.' 0.1 ' ' 1
## 
## Residual standard error: 0.3809 on 19 degrees of freedom
##   (1 observation deleted due to missingness)
## Multiple R-squared:  0.03237,    Adjusted R-squared:  -0.01855 
## F-statistic: 0.6357 on 1 and 19 DF,  p-value: 0.4351
```

```
fm.c <- lm(dprime ~ pcu_score, data=gamePerf.wmc[gamePerf.wmc$age_grp == 'C',])
summary(fm.c)
```

```
## 
## Call:
## lm(formula = dprime ~ pcu_score, data = gamePerf.wmc[gamePerf.wmc$age_grp == 
##     "C", ])
## 
## Residuals:
##      Min       1Q   Median       3Q      Max 
## -0.74446 -0.32702 -0.08629  0.27725  1.06990 
## 
## Coefficients:
##             Estimate Std. Error t value Pr(>|t|)    
## (Intercept)   1.0450     0.1629   6.417 2.49e-07 ***
## pcu_score     0.2431     0.4835   0.503    0.618    
## ---
## Signif. codes:  0 '***' 0.001 '**' 0.01 '*' 0.05 '.' 0.1 ' ' 1
## 
## Residual standard error: 0.4558 on 34 degrees of freedom
## Multiple R-squared:  0.007381,   Adjusted R-squared:  -0.02181 
## F-statistic: 0.2528 on 1 and 34 DF,  p-value: 0.6183
```

# Information on the clip durations

```
clip.durs <- unique( answers.game.final[c("clip_id", "src_stop_time", "locationsNum", "targetsNum", "emptyNum")] )
# descriptive statistics
describe(clip.durs$src_stop_time)
```

```
## clip.durs$src_stop_time 
##       n missing  unique    Info    Mean     .05     .10     .25     .50 
##      30       0      30       1   11.47   5.770   5.981   6.974  10.706 
##     .75     .90     .95 
##  14.757  18.105  18.948 
## 
## lowest :  4.806  5.739  5.807  6.000  6.007
## highest: 17.918 18.100 18.152 19.600 24.193
```

```
watchingTime <- sum(clip.durs$src_stop_time) # in seconds
watchingTime / 60 # 5 minutes
```

```
## [1] 5.733784
```

```
(watchingTime / 60 - 5) * 60 # 44 secons
```

```
## [1] 44.02703
```
